# Enzyme-Responsive Zr-Based Metal–Organic Frameworks for Controlled Drug Delivery: Taking Advantage of Clickable PEG-Phosphate Ligands

**DOI:** 10.1021/acsami.3c03230

**Published:** 2023-05-30

**Authors:** Carolina Carrillo-Carrión, Valentine Comaills, Ana M. Visiga, Benoit R. Gauthier, Noureddine Khiar

**Affiliations:** † Institute for Chemical Research (IIQ), CSIC-University of Seville, 41092 Sevilla, Spain; ‡ Andalusian Center for Molecular Biology and Regenerative Medicine (CABIMER), Junta de Andalucía-University of Pablo de Olavide-University of Seville-CSIC, 41092 Sevilla, Spain; § Centro de Investigación Biomédica en Red de Diabetes y Enfermedades Metabólicas Asociadas (CIBERDEM), 28029 Madrid, Spain

**Keywords:** metal−organic frameworks, biocompatibility, enzyme-responsive, controlled drug-release, click chemistry

## Abstract

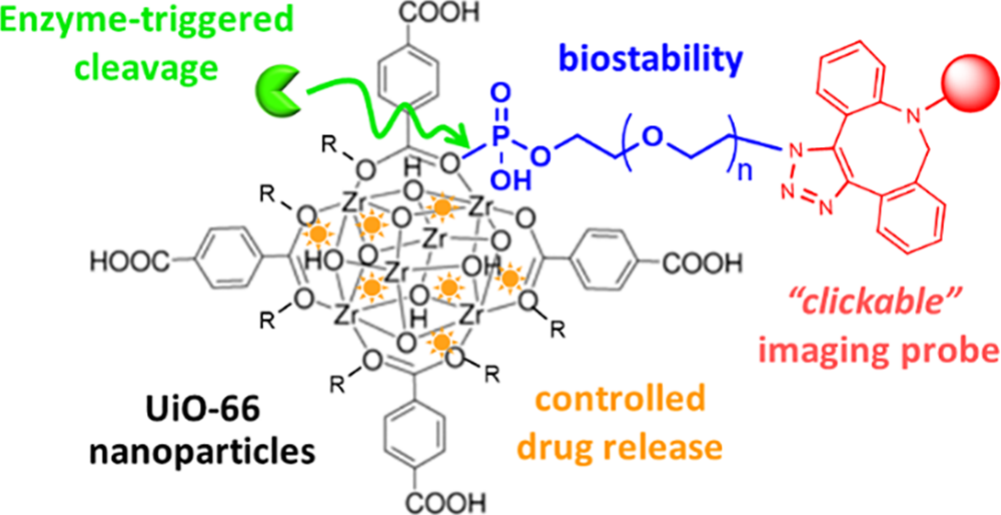

We report for the first time the controlled drug release from a nanoscale Zr-based metal–organic framework (MOF), UiO-66, in the presence of the enzyme alkaline phosphatase (ALP). This unprecedented reactivity was possible thanks to the prior functionalization of the MOF with N_3_–PEG–PO_3_ ligands, which were designed for three specific aims: (1) to impart colloidal stability in phosphate-containing media; (2) to endow the MOF with multifunctionality thanks to azide groups for the covalent attachment of an imaging agent by click-chemistry; and (3) to confer stimuli-responsive properties, specifically the selective release of doxorubicin triggered by the enzymatic activity of ALP. Cell studies revealed that the functionalization of the MOF with N_3_–(PEG)_20_–PO_3_ ligands improved their intracellular stability and led to a sustained drug release compared to the bare MOF. More importantly, an enhanced drug release was observed in cells with higher expression of ALP genes (HeLa versus MDA-MB-231 and MCF7), confirming the ALP-responsiveness of the system inside living cells.

## Introduction

The number of people diagnosed each year with chronic diseases (cancer, cardiovascular diseases, diabetes, autoimmune or neurodegenerative diseases, among others) is continuously increasing. According to the World Health Organization (WHO), early detection, screening, and efficient treatment are the key components for an effective response to human diseases.^1^ Within this context, the design of more effective and selective drug delivery systems (DDS) for therapy, which can also integrate bioimaging function for detection and monitoring, giving rise to theranostic platforms is of utmost importance.

Nanosized metal–organic frameworks (MOFs), consisting of metal ions or clusters coordinated by multidentate organic ligands,^2^ have recently emerged as promising drug nanocarriers,^3^ due to their particular physicochemical properties, such as hybrid organic–inorganic nature, high and tunable porosity, exceptional drug loading capacity, and ease of functionalization. These features allow the rational design of various types of efficient and “smart” DDS, based on the specific needs of the intended application. Despite this, the limited stability of many MOFs under biological settings (i.e., aqueous solutions, high ionic strength, presence of phosphate ions, etc.) hampers their path to practical clinical applications. In the particular case of Zr-MOFs (e.g., UiO-66, PCN-222, or NU-1000), which have been widely investigated as DDS,^4^ they are stable in water and at acidic pH but degraded very fast in phosphate media due to the competition of phosphate species for the Lewis metal centers (Zr). The functionalization of the outer surface of MOF particles with polymers, commonly polyethylene glycol (PEG) and other amphiphilic polymers, has already been applied to different types of MOFs (e.g., ZIF-8, MIL-101(Fe), and UiO-66) and has been demonstrated to be an easy and effective approach to improve not only the colloidal stability but also the biocompatibility, cellular uptake, circulation time, and pharmacokinetic drug profile.^4−10^ Focusing on Zr-MOFs, Fairen-Jimenez and co-workers reported a Cu(I)-catalyzed click chemistry approach to functionalize UiO-66 with PEG moieties.^10^ The same group demonstrated that using a PEGylation approach with phosphate-modified methoxy polyethylene glycol (mPEG–PO_3_), the dried nanosized Zr-MOFs (i.e., lyophilized) could be completely redispersed in water, avoiding the common aggregation issue that prevented the storage of these MOFs in dry form. This approach relies on the strong binding affinity between Zr and phosphate, which was previously utilized to functionalize nanoMOFs with phospholipid bilayers,^11^ or phosphate-modified oligonucleotides/DNA.^12,13^ This methodology effectively shields Zr-MOFs from potential phosphate ion attacks.

In addition to achieving the biostability of MOFs, the incorporation of additional functionalities such as imaging or targeting agents^14^ or the coencapsulation of various therapeutic agents into a single MOF particle via pore space partitioning approaches^15^ is being intensively investigated. Multifunctional MOFs, capable of integrating imaging and treatment strategies into an “all-in-one” nanosystem, are the key for the next-generation of MOF-based theranostic platforms.^14^ To date, there are three main approaches for the construction of MOFs with additional functionalities/capabilities: (i) the encapsulation or loading of the functional molecules within the pores of MOFs, (ii) their incorporation as intrinsic structural components of MOFs, or (iii) their attachment to the MOF surface by covalent/electrostatic/coordinative conjugation. The latter approach is the most advantageous for targeting agents (such as antibodies, aptamers, or small molecules), as they must be situated on the outer surface and be easily accessible. Since these strategies involve careful optimization on a case-by-case basis, the development of a versatile method applicable for all MOF types, or at least a subfamily of them, would be appealing. In this direction, the use of “click chemistry” can play a major role considering their beneficial features such as high selectivity, high efficiency/yield, and fast reaction rate. Clickable MOFs have been prepared so far by using de novo synthetic approaches, which means that the azide or alkyne groups are incorporated during the MOF formation either using azide/alkyne-bearing organic linkers,^16^ azide-bearing acid as modulators,^10,17^ or amino-bearing linkers with the subsequent −NH_2_ conversion into the −N_3_ group.^18,19^ These strategies, although successful in some cases, may encounter ligand solubility problems and may also affect the structural integrity of the MOF or its porosity. To remedy these caveats, we present herein the design of a bifunctional PEG ligand, specifically N_3_–(PEG)_20_–PO_3_, which allows the incorporation of −N_3_ groups together with the MOF functionalization with the polymer through an easy and reproducible one-pot procedure.

On the other hand, much effort is being focused on designing stimuli-responsive MOFs for the controlled release of drugs upon the presence of specific chemical and physical triggers.^14,19^ There are two general pathways to this: one involves the triggered degradation of the MOF particles and the release of the loads, while the other consists of the surface functionalization of the MOFs yielding stimuli-responsive gates that can be unlocked in the presence of appropriate triggers. Chemical triggers, such as pH changes, ions, or redox agents, and physical ones (e.g., light or heat), are commonly used. However, biological triggers such as enzymes, DNAzymes, and miRNAs are much less investigated and exploited.^20^ Herein, taking advantage of the phosphate–Zr coordinative bond used for the functionalization of the Zr–MOFs with the designed N_3_–(PEG)_*n*_–PO_3_ polymer, we propose a general approach to generate alkaline phosphatase (ALP)-responsive Zr-MOF nanoparticles that allow the controlled release of drugs, previously loaded within the MOF pores, under the presence of the enzyme ALP. This enzyme is often overexpressed in various cancers (e.g., pancreatic, prostate, colon, lung, gastric, and osteosarcoma cancers)^21,22^ and other human pathologies such as liver diseases^23^ and osteoblast dysfunction,^24^ making it an important clinical marker. Furthermore, it has more recently been recognized as a target enzyme for the development of improved therapies.^25^ Therefore, the design of ALP-responsive drug delivery systems would be advantageous for the treatment of these cancers in a selective manner.

**Scheme 1:**
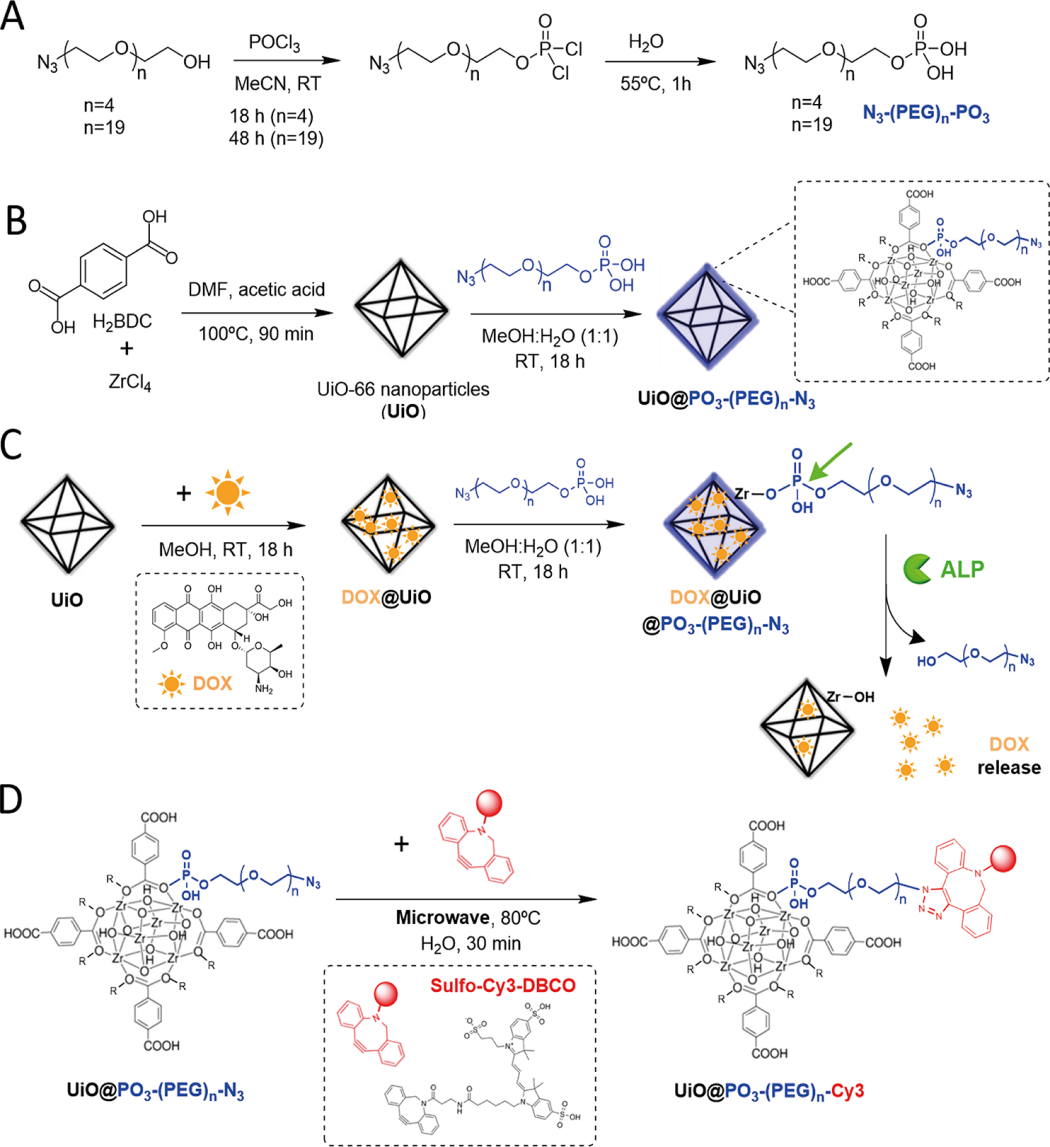
(A) Simplified Scheme of the Synthetic Route to N_3_–(PEG)_*n*_–PO_3_ Ligands; (B) Schematic Illustration of the Solvothermal Synthesis of UiO-66 MOF Nanoparticles (UiO) and the Following Postsynthetic Functionalization Strategy To Coat the UiO with N_3_–(PEG)_*n*_–PO_3_ Ligands; (C) Representation of the Loading of UiO Nanoparticles with Doxorubicin (DOX), Followed by Their Surface Functionalization with the Ligands, and the Proposed DOX Release Triggered by the Enzyme Alkaline Phosphatase (ALP); (D) Scheme of the Attachment of a Fluorescent Probe (Sulfo-Cy3-DBCO) to the Surface of the UiO@PO_3_–PEG–N_3_ Particles by Using a Copper-Free Microwave-Assisted “Click” Reaction

With these challenges in mind, we report herein the synthesis of N_3_–(PEG)_*n*_–PO_3_ ligands (*n* = 5 and 20) and develop a one-step and efficient method to functionalize the nanosized UiO-66 MOFs with these polymers, resulting in clickable multifunctional UiO-66 nanoparticles with ALP-responsive capability for the controlled release of a drug (doxorubicin as model antitumor drug). The multifunctionality is demonstrated by performing a postsynthetic modification of the UiO@PO_3_–PEG–N_3_ nanoparticles with a fluorescent molecule (Cy3 as the model imaging probe) by using a high-yielding copper-free “click” reaction compatible with the cargo-loaded MOFs (Scheme 1).

## Results and Discussion

### Design and Synthesis of Clickable PEG-Phosphate Ligands

Clickable PEG-Phosphate ligands were designed to have the following key structural features: (1) a polyethylene glycol chain to provide hydrophilicity and biocompatibility, as well as to prevent opsonic interaction and macrophage uptake in future in vivo studies;^26^ (2) an azide (−N_3_) group to allow the further modification with alkyne-bearing molecules by click reaction; and (3) a phosphate group (−PO_3_) to form strong Zr–O–P coordination bonds between the zirconium sites on the MOF surface and the PEG ligands. We prepared N_3_–(PEG)_*n*_–PO_3_ ligands with two different well-defined PEG chain lengths, in particular *n* = 5 (MW = 343) and *n* = 20 (MW = 1004), in order to study the effect of the PEG length on the colloidal stability of the resulting MOFs. The synthetic route consisted of a SN2-type reaction between the monohydroxy-PEG derivatives modified with an azide functionality (obtained in two steps from the starting diol)^27^ with phosphorous oxychloride (POCl_3_), followed by a hydrolysis step (Scheme 1A). In the first reaction step, the longer PEG (*n* = 20) showed a slower kinetic than the short-PEG (*n* = 5), requiring a longer reaction time to achieve high conversion (>80%). It is worth noting that this reaction is extremely sensitive to experimental conditions. Under certain conditions, a dimeric derivative resulting from the attack of a second PEG molecule on the dichloride intermediate with two leaving groups may form. These conditions include working with concentrated solutions, large excess and fast addition of PEG, or using high temperature and long reaction times (Scheme S1). Therefore, careful control of these parameters is crucial to ensure successful synthesis of the desired PEG derivative without unwanted side reactions. Under optimized conditions (see the ESI for details), the selectivity toward the target ligands was 94% (for *n* = 5) and 83% (for *n* = 20) as determined by high-performance liquid chromatography (HPLC). ^1^H and ^31^P NMR spectroscopy, high-resolution electrospray ionization-mass spectrometry (ESI-MS), and HPLC-MS confirmed the formation and purity of N_3_–(PEG)_5_–PO_3_ and N_3_–(PEG)_20_–PO_3_ (Figures S1–S4).

### Synthesis and Characterization of MOF Nanoparticles

**Figure 1:**
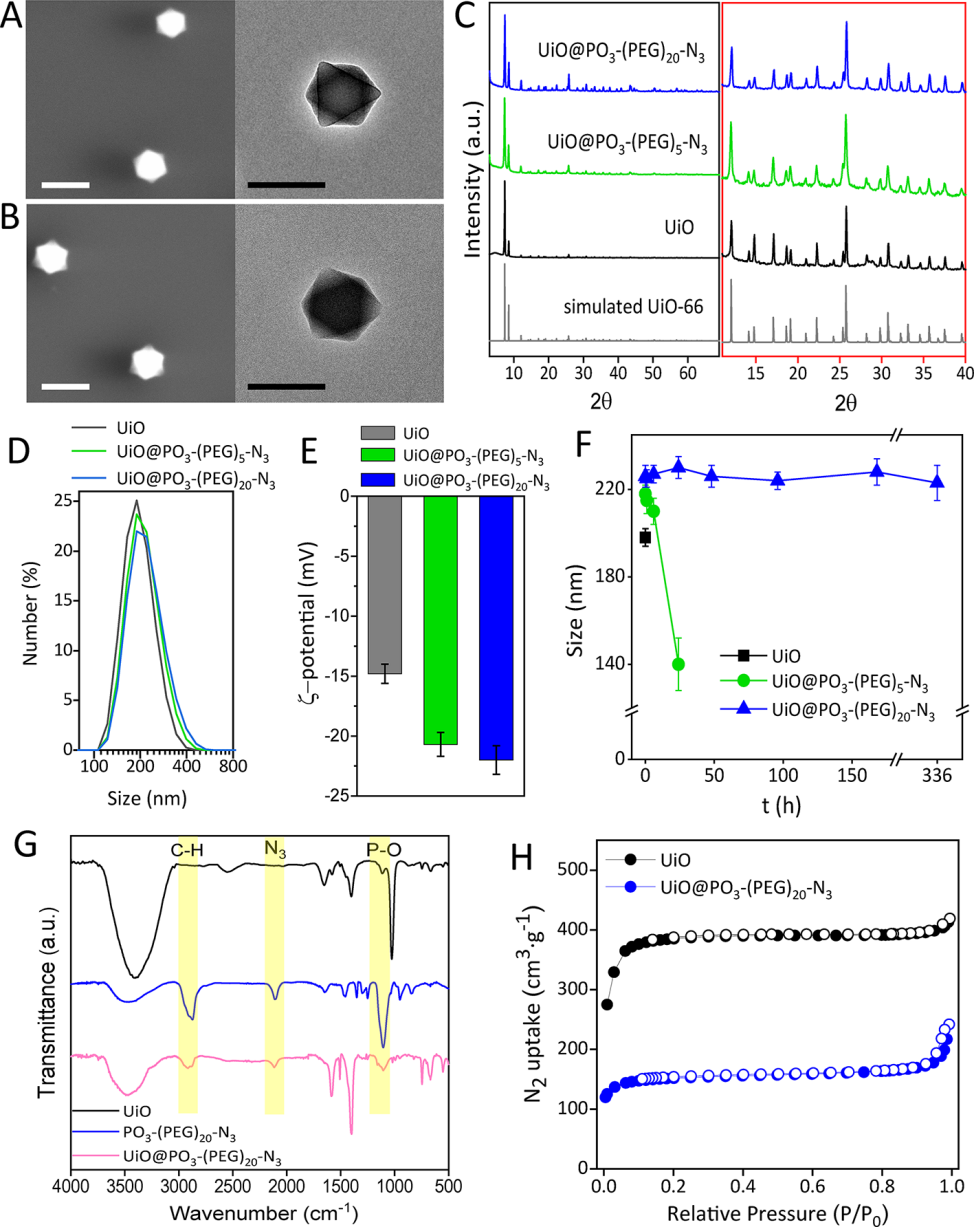
(A) Characterization of UiO nanoparticles before and after their functionalization with N_3_–(PEG)_*n*_–PO_3_ ligands. (A,B) Representative SEM (left) and TEM (right) images of UiO (A) and UiO@PO_3_–(PEG)_20_–N_3_ particles (B). Scale bars correspond to 200 nm. (C) PXRD patterns of the as-prepared UiO and UiO@PO_3_–(PEG)_*n*_–N_3_ (*n* = 5 and 20) particles, and the simulated UiO-66 calculated from cif.file (COD (Crystallography Open Database): 4512072) for comparison. Magnification of the 2θ range between 11° and 40° is also shown to better visualize the peaks. (D) Dynamic light scattering (DLS) size distributions by number of UiO and UiO@PO_3_–(PEG)_*n*_–N_3_ (*n* = 5 and 20) particles dispersed in Milli-Q water. (E) ζ-potential of the same water dispersions of the particles. (F) Colloidal stability over time of the particles dispersed in PBS, as determined by DLS. (G) FT-IR spectra of UiO, N_3_–(PEG)_20_–PO_3_, and UiO@PO_3_–(PEG)_20_–N_3_. (H) N_2_ isotherms (77 K) of UiO and UiO@PO_3_–(PEG)_20_–N_3_ particles. Closed symbols represent adsorption, and empty symbols represent desorption.

As a representative Zr-based MOF, UiO-66 (UiO stands for Universitetet i Oslo)^28^ was selected due to the following reasons: (i) it can be easily synthesized at the nanometric scale, and the synthetic method is low-cost and scalable,^29^ (ii) it has good biocompatibility and low toxicity,^30^ and (iii) its cellular internalization mechanism is well studied.^31^ UiO-66 nanoparticles (in the following referred to as UiO) were synthesized under solvothermal conditions, using acetic acid to modulate the crystallite size, resulting in octahedral nanoparticles with an average size (edge length) of ∼180 nm under optimized reaction time (90 min) as determined by scanning electron microscopy (SEM) and transmission electron microscopy (TEM) (Figures 1A and S5A). The reaction time (crystal growth time) had a notable effect on the morphology (size and shape) of the UiO-66 particles. Short times (45 min) led to quasi-spherical particles of ca. 100 nm, while long reaction times (24 h) resulted in a mixture of octahedral and tetragonal pyramidal particles with sizes of ca. 250 nm (Figure S6). Powder X-ray diffraction (PXRD) revealed that as-prepared UiO nanoparticles were highly crystalline and displayed the characteristic Bragg peaks of UiO-66, which are well in accordance with the simulated XRD pattern (Figure 1C). Next, we performed the surface functionalization of the particles with both previously prepared N_3_–(PEG)_*n*_–PO_3_ ligands (*n* = 5 and 20). In a typical MOF-PEG functionalization experiment, an excess of ligand was added to a colloidal suspension of UiO particles and subsequently incubated it at room temperature (RT) under gentle stirring (see the ESI for details). Afterwards, the nanoparticles were purified by centrifugation, washed with water, and finally redispersed in either MeOH or water. We denote these functionalized nanoparticles as UiO@PO_3_–(PEG)_*n*_–N_3_ (*n* = 5 or 20, depending on the PEG ligand used). To optimize the functionalization method, experimental parameters such as the solvent and the ligand/MOF ratio were studied in order to maximize the functionalization efficiency (Table S1). This efficiency was determined by HPLC quantification of the nonbound ligands (remaining in the supernatant after purification of the particles by centrifugation); see the ESI for details. Under optimized conditions (1 μmol of N_3_–(PEG)_*n*_–PO_3_ ligand per mg of UiO particles, MeOH:H_2_O (1:1), and 18 h of incubation time), the functionalization efficiencies were 87 and 74% for *n* = 5 and 20, respectively. Therefore, the amount of incorporated N_3_–(PEG)_*n*_–PO_3_ ligands in the UiO@PO_3_–(PEG)_*n*_–N_3_ particles was 0.87 μmol/mg (23.0 wt %) for *n* = 5 and 0.74 μmol/mg (42.6 wt %) for *n* = 20, as determined by HPLC. Additionally, we measured the ratio of P to Zr by using inductively coupled plasma-optical emission spectroscopy (ICP-OES, Table S2) to estimate the wt% of PEG in the MOF particles, obtaining values of 19.0 wt % for *n* = 5 and 38.4 wt % for *n* = 20. In addition, comparison of the thermogravimetric analysis (TGA) curves of the particles before and after functionalization allowed also to determine the amount of N_3_–(PEG)_20_–PO_3_ ligands in the particles, which turned out to be ca. 32 wt % (Figure S7). Importantly, the PEG content determined by all the characterization techniques was in the same order of magnitude.

The as-prepared UiO@PO_3_–(PEG)_*n*_–N_3_ particles were then characterized by different complementary techniques to further demonstrate the successful attachment of the ligands to the MOF surface. SEM and TEM images (Figures 1B and S5B) and PXRD (Figure 1C) verified that the shapes and crystallinity of the MOF particles were mostly preserved after the ligand functionalization. Careful inspection of the TEM images revealed some particles with edges slightly worn after the attachment of PEG ligands. Moreover, TGA and DSC curves of the UiO@PO_3_–(PEG)_20_–N_3_ particles (Figure S7) showed a distinct endothermic peak between 450 and 530 °C, which is assigned to the decomposition of UiO-66, thus confirming that the crystalline structure of the MOFs is maintained in spite of the functionalization with the N_3_–PEG–PO_3_ ligands, in accordance with PXRD results. TEM coupled with energy-dispersive X-ray spectroscopy (EDX) analysis was used to qualitatively analyze the composition of the functionalized particles, showing a strong peak corresponding to Zr atoms as well as the presence of P and N elements coming from the ligands (Figure S8). It is worth noting that reliable quantification by TEM-EDX was not possible as the lines corresponding to the P and Zr elements (the Lα line for Zr is 2.042 keV and the Kα line for P is 2.012 keV) cannot be adequately separated by EDX spectroscopy. DLS measurements of the functionalized particles showed that the hydrodynamic sizes were slightly larger than that of the bare UiO particles, having sizes of 215 ± 3 nm (for PEG length *n* = 5) and 224 ± 5 nm (for PEG length *n* = 20) compared to 198 ± 4 nm (for bare particles), Figure 1D. Notably, the particles were highly homogeneous as indicated by the low polydispersity index (Table S3). Moreover, the ζ-potential of the UiO particles, which are negatively charged in MilliQ water (pH ∼ 6.5), became more negative after their functionalization with the PEG ligands, from −14.8 ± 0.8 mV for the bare particles to −20.7 ± 1.0 mV and – 22.0 ± 1.2 mV for the UiO@PO_3_–(PEG)_5_–N_3_ and UiO@PO_3_–(PEG)_20_–N_3_ particles, respectively (Figure 1E). This change in the surface charge was attributed to the attachment of terminal −PO_3_ groups to the unsaturated Zr cations on the UiO surface. To study the colloidal stability over time of the UiO particles before and after the PEG functionalization in a relevant biological medium (PBS, 0.1 M, pH = 7.4), we monitored changes in the hydrodynamic size by DLS (Figure 1F and Table S4). As expected, the bare UiO particles degraded very fast due to the attack of the phosphate ions to the Zr sites on the particle. By simple visual inspection, one could see how the characteristic turbidity of the MOF particles gradually disappears within a few minutes. When UiO was modified with the short PEG ligand, i.e., N_3_–(PEG)_5_–PO_3_, the particles were stable only for several hours, indicating that the short PEG chain was unable to completely prevent access and attack of the phosphate ions to the uncoordinated Zr sites on the MOF surface. In contrast, the longer N_3_–(PEG)_20_–PO_3_ ligand endowed the particles with long-term stability, at least up to 2 weeks as measured. These results disclosed thus the strong impact of the PEG chain length for stabilizing the UiO particles. The structural integrity of UiO@PO_3_–(PEG)_20_–N_3_ particles was also confirmed by PXRD of the particles after incubation in PBS for 1 week (Figure S9). Because of that, the following experiments were only performed with the most stable UiO@PO_3_–(PEG)_20_–N_3_ particles. Fourier transform infrared (FT-IR) spectra (Figure 1G) provided further evidence of the incorporation of N_3_–(PEG)_20_–PO_3_, as indicated by the appearance of three new bands at 2800–2900 cm^–1^, at around 2100 and 1100 cm^–1^, which were attributed to the stretching vibrations of C–H, N_3_, and P–O, respectively, from the PEG molecules. Additionally, we conducted N_2_ uptake experiments to determine the effect of the functionalization on the porosity of the MOF particles. Although the isotherms showed in principle a large decrease in the area after the surface modification of the UiO particles with the N_3_–(PEG)_20_–PO_3_ ligands (Figure 1H), the actual Brunauer–Emmett–Teller area (*S*_BET_) and micropore volume (*V*_micro_) of the UiO@PO_3_–(PEG)_20_–N_3_ particles were not so different from those obtained for pristine UiO particles after correction considering exclusively the UiO weight (Table S5). Even when a partial pore-blocking after PEG functionalization cannot be completely ruled out, as this effect has been previously reported for other polymer-functionalized MOFs,^4,11^ the data revealed that a significant fraction of the pores remained accessible.

To further verify the modification of MOF particles with N_3_–PEG–PO_3_ ligands and have some indications regarding the interaction between these ligands and the MOF framework, we performed ^1^H and ^31^P nuclear magnetic resonance (NMR) analyses (Figure S11). We did first the NMR of a stable dispersion of the UiO@PO_3_–(PEG)_20_–N_3_ particles in deuterated water (D_2_O), where the ^1^H NMR spectra clearly showed the characteristics peaks of the N_3_–(PEG)_20_–PO_3_ molecules. We also observed signals at 8.3 ppm, attributed to the uncoordinated carboxylic groups on the MOF surface from the organic linkers (H_2_BDC). Compared with the ^1^H NMR spectra of free N_3_–(PEG)_20_–PO_3_ (Figure S2A), we observed the broadening and splitting of some signals. The splitting or change in chemical shifts is due to the fact that some H atoms, which are equivalent in the free PEG molecules, are located in different chemical environments once attached to the MOF surface. The broadening of the signals, on the other hand, is the consequence of restricted molecular motion and slower tumbling of the small molecule once attached to the MOF particles. We noted the absence of the phosphorus resonance in the ^31^P NMR spectra of UiO@PO_3_–(PEG)_20_–N_3_ particles, while the free N_3_–(PEG)_*n*_–PO_3_ presented a sharp peak at around 0 ppm (Figures S1B and S2B). Next, the particles were digested by treatment with a concentrated NaOH solution, and we recorded the NMR spectra of the resulting mixture solution. The appearance of the phosphorus peak at 0 ppm, together with the appearance of the signal at 7.8 ppm from the BCD (linkers of the UiO-66 particles), revealed the dissolution of the UiO nanoparticles and the consequent detachment of the PEG ligands from the MOF surface. These results suggest that the binding of the ligands to the MOFs takes place through the coordination of the terminal −PO_3_ of the PEG molecules with the Zr sites at the surface of the UiO, which is consistent with previously reported studies.^11−13^

### Drug Loading and Enzyme-Responsive Properties of MOF Nanoparticles

**Figure 2:**
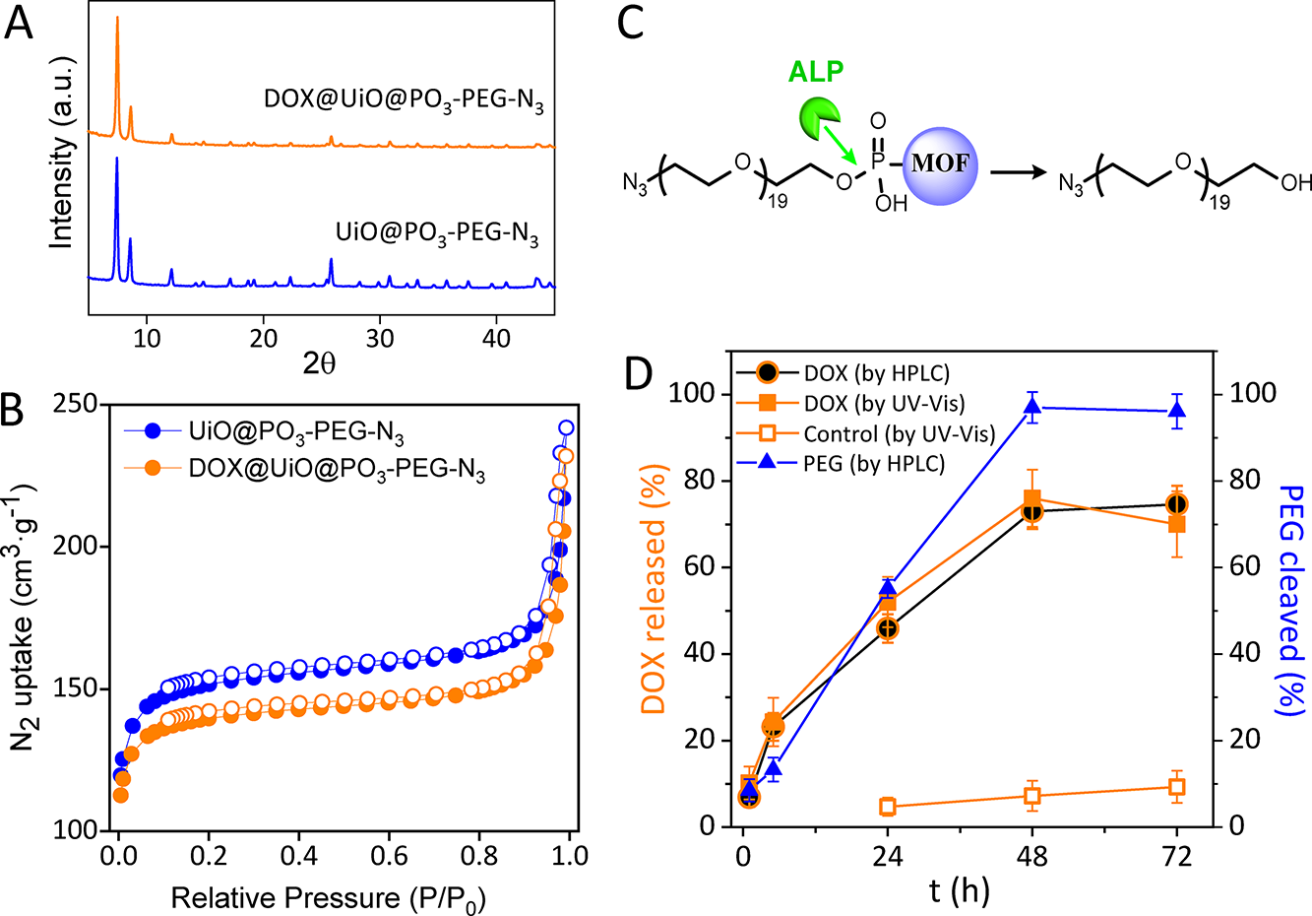
(A) PXRD of UiO@PO_3_–PEG–N_3_ particles without and with DOX molecules loaded on the MOF particles. (B) N_2_ isotherms (77 K) of particles without and with DOX loaded. Closed symbols represent adsorption, and empty symbols represent desorption. (C) Schematic illustration of the cleavage of the phosphate ester bond in the DOX@UiO@PO_3_–PEG–N_3_ particles by ALP enzymolysis, indicating the cleavage site on the basis of the MS data. (D) In vitro kinetic profiles of DOX released and PEG cleaved from the MOF particles (5 mg/mL) exposed to ALP (1 U/mL) in HEPES buffer (0.01 M, 2 mM Mg^+2^, pH = 7.2) at 37 °C. Data from HPLC-MS and UV–vis analyses are given. As a control, the profile of DOX released from MOF particles in the absence of ALP is also shown (Control); DOX was not detected (below the limit of detection of the UV–vis method) at times of 1 and 5 h.

With these stable UiO@PO_3_–(PEG)_20_–N_3_ particles at hand, we sought to explore whether they were sensitive to the presence of the enzyme ALP. If so, enzymatic cleavage of the phosphate ester group between the PEG ligand and the MOF surface would facilitate the triggered release of drugs loaded onto the MOF selectively within ALP-overexpressing cancer cells/tissues. To test this hypothesis (Scheme 1C), we first loaded the UiO particles with the chemotherapy drug DOX (see the ESI for details) and the DOX@UiO particles were further modified with N_3_–(PEG)_20_–PO_3_ as previously described, resulting in DOX@UiO@PO_3_–PEG–N_3_ particles. The loading step was performed prior to PEG functionalization of the particles since we obtained a very low DOX loading (less than 1 wt %) when performing the drug encapsulation on the functionalized UiO@PO_3_–PEG–N_3_ particles. Taking into account that significant N_2_ uptake is still possible in the PEGylated particles as discussed above, the low DOX loading once the UiO surface has been modified with PEG ligands is likely due to blocking of surface adsorption sites by coordination of PEG to Zr clusters (with associated steric hindrance) rather than pore openings not being accessible. By first performing the DOX loading, we would ensure that the drug molecules are actually loaded onto the MOF particle and not simply adsorbed or trapped between the PEG chains. The drug loading efficiency and loading capacity were 11 and 5.5 wt %, respectively, as determined by UV–Vis spectroscopy (Figure S9). The amount of DOX lost during the following PEGylation process on the DOX@UiO particles was only 0.21 wt %, being therefore the final content of DOX in the DOX@UiO@PO_3_–PEG–N_3_ particles of 5.3 wt %. This low loading capacity, comparable with values obtained for DOX adsorbed on the surface of solid particles, is in line with the fact that DOX is too big (15.3 × 11.9 Å)^32^ to penetrate the porosity of UiO-66. Note that the UiO-66 contains small micropores (∼11 Å octahedral and ∼8 Å tetrahedral), and the pore window through which adsorbates can access this porosity is even smaller, which should preclude any significant pore loading of DOX. The hydrodynamic size and ζ-potential of the DOX@UiO and DOX@UiO@PO_3_–PEG–N_3_ particles were very similar to those of the nonloaded counterparts, which did not allow inferring clues about the localization of the DOX (Table S6 and Figure S13), but importantly the DOX loading did not affect the crystallinity of the particles as determined by PXRD (Figure 2A). The BET area showed only a small decrease from 511 to 470 m^2^·g^–1^ due to the DOX loading (Figure 2B), which is consistent with the addition of non-porous DOX mass (5.3 wt %) as shown in Table S7 after correction of the data by subtracting such contribution. Based on these findings, we can conclude that the DOX is most likely located on the UiO surface (i.e., surface loading), which is in agreement with a recently published study about the location of DOX (pore or surface) on different types of MOFs.^32^

We next incubated the UiO@PO_3_–PEG–N_3_ particles with ALP in HEPES buffer at 37 °C for different times, observing the successful cleavage of the phosphate ester bond and subsequent release of DOX, as confirmed by HPLC-MS. Careful analysis of the MS spectrum (Figure S15) disclosed the specific cleavage site (Figure 2C), since the N_3_–(PEG)_20_–OH (MW = 923.5) is the molecule released to the medium, having a molecular ion *m*/*z* [M + H_2_O]^+^ = 941.8 under positive ion mode. Figure 2D depicts the time-dependent release of DOX from the particles upon subjecting the MOF particles (5 mg/mL) to ALP (1 U/mL), and analyzed by both HPLC-MS and UV–Vis spectroscopy. Both techniques led to very similar kinetic profiles, ensuring the reliability of the data. We also measured the PEG ligand cleaved over time by HPLC. Percentage data were determined by quantifying the total content of DOX and PEG ligand in the particles by digestion with NaOH. We noted that the DOX release reached a saturation value of ∼73% after 48 h, while the cleavage of PEG was quantitative. This seems to indicate that some DOX molecules are strongly adsorbed on the MOF framework, which hinders their release. This lack of complete release could be also indicative of surface binding rather than pore loading.^32^ For comparison, we measured also the release of DOX from the particles in the absence of ALP, resulting in less than 10% after 72 h (determined by UV–Vis). This clearly indicated that the PEG coating efficiently prevents the premature drug release from the UiO nanoparticles.

To gain further insight into the mechanism of release, we subjected the DOX@UiO@PO_3_–PEG–N_3_ particles to different pH values, covering the range from 5.5 to 7.4. Investigating the behavior of the as-designed MOF particles under various biologically relevant pHs is quite important given the fact that the pH in tumor and inflammatory tissues tends to be more acidic, being close to 5.5 in cancer cells, whereas the pH in blood and healthy tissue is ∼7.4. After incubation of the particles in PBS at three different pH values (pH = 7.4, 6.5 and 5.5), the DOX release profiles were determined by UV–Vis spectroscopy (see the ESI for details). Results revealed a slightly higher DOX release rate at acidic condition (pH 5.5, 18.7%) than those at the neutral condition (pH 7.4, 11.3%) in 72 h, as is shown in Figure S14A. It is worth noting that we observed a rapid release of DOX in the first hours, and no significant differences were found at longer times, which is in agreement with already published data with similar MOF particles.^10,32^ This is most likely attributed to the fact that DOX is attached to the external surface of the UiO particles, consistent with the findings discussed above. Interestingly, the effect of pH was quite small compared to the presence of the ALP enzyme (Figure S14B), pointing out the selectivity of the as-designed drug delivery system toward ALP activity and confirming that DOX release is due to enzymatic cleavage of the phosphate ester bond between the MOF surface and the PEG coating, at least in the time scale studied (up to 72 h).

### Click Chemistry on MOF Nanoparticles

In order to validate that the terminal azide group of the N_3_–(PEG)_20_–PO_3_ ligand was accessible and reactive once attached to the MOF surface, we performed a copper-free click reaction to modify the functionalized DOX@UiO@PO_3_–PEG–N_3_ particles with a fluorescent probe (Sulfo-Cy3-DBCO), Scheme 1D. Importantly, the use of a strain-promoted alkyne–azide cycloaddition (SPAAC) reaction here ensured high efficiency covalent modification without the need of toxic metals, i.e., copper as a catalyst, with the consequent advantage for the further application of these particles to biological systems. The click reaction was optimized and promoted by microwave irradiation, aimed at having a high yield (efficiency = 98%, as determined by UV–Vis spectroscopy, Figure S16) in only 30 min (see the ESI for details). DLS and LDA measurements after the dye modification did not show significant changes, but notably the PDI was still quite low pointing to a fairly narrow size distribution of the dye-modified MOF particles (Table S8 and Figure S17). Although the small increase in the hydrodynamic size (by about ∼4 nm) is not significant to draw a conclusion, it did allow us to rule out the formation of aggregates during the SPAAC reaction and it was indicative of the good dispersibility of the particles after their modification. Furthermore, the Cy3-modified MOF particles presented a very similar thermal stability to the nonmodified particles as determined by TGA (Figure S17), which further confirmed that the MOF structure was not compromised during the SPAAC reaction. Notably, the Cy3 content derived from comparison of the TGA curves was determined to be 3.2 wt % (Figure S18), which is in close agreement with the quantitative data calculated by UV–Vis spectroscopy (3.5 wt %). These results indicate that the functionalization of the UiO particles with N_3_–PEG–PO_3_ ligands did not only improve the stability in phosphate-containing media, but also provided further reactive sites for the covalent attachment of alkyne/DBCO-containing functional molecules, such as fluorescent agents (as demonstrated here) but also applicable to tumor-targeting molecules.

### Evaluation of MOF Nanoparticle Performance in Living Cells

To assess whether the integrity of the UiO@PO_3_–PEG–N_3_ particles was preserved during cellular uptake, in other words, whether the PEG ligands could detach from the particle surface in the cell media and/or during internalization, we performed experiments with doubly fluorescent-labeled MOF nanoparticles to easily monitor the particles by confocal microscopy and flow cytometry. To this end, a bright green-fluorescent dye, specifically fluorescein (F) was loaded within the pores of UiO before PEG functionalization (in the same way as described above for DOX). Next, these particles were functionalized with the PEG ligands and further modified with Cy3 by click reaction (using already optimized procedures in both steps), finally obtaining F@UiO@PEG-Cy3 particles. Note that the reason for using fluorescein as the fluorescent cargo here instead of DOX is because the fluorescence spectrum of DOX and Cy3 cannot be separated under the fluorescence microscope. Fluorescent F@UiO particles were used as control particles to study the effect of the PEG coating on the cargo release kinetics. To first asses the stability of the PEGylated MOFs in cell culture medium, the UiO@PEG-Cy3 particles were incubated at 37 °C in complete cell medium (DMEM supplemented with 10% FBS, see the ESI for details) and the amount of Cy3 released after different incubation times (3, 10, 24, and 48 h) was determined by UV–Vis spectroscopy. Cy3 was not detected at short incubation times, requiring 48 h to observe a release of only 3.4% (calculated with respect to the total Cy3 present in the particles; Figure S19). This finding allowed us to conclude that the covalent binding of the Cy3-DBCO dye to PEG-N3 on the surface of MOF is resistant to the culture medium, and therefore, in the absence of any type of cellular activity, nonspecific detachment of the dye is not expected.

To select next the appropriate concentration of MOF nanoparticles, we performed viability assays based on ATP measurements in HeLa cells after exposure to the different MOF particles: UiO, F@UiO, UiO@PEG-Cy3 and F@UiO@PEG-Cy3. All particles showed the expected characteristic dose-dependent response (Figure S20A), albeit, the doubly fluorescent-labeled particles exhibiting slightly more cytotoxicity (see values of half-maximal effective concentrations EC_50_ determined from the viability curves). Based on these data, we selected a concentration of 0.2 mg/mL as the optimal for the following uptake studies in order to achieve the highest cell response (maximum dose) while maintaining a viability greater than 80%. Besides, we investigated the toxicity of these MOF particles to normal cells at this working concentration, selecting human mesenchymal stem cells (MSCs) as a prototypic of adult stem cell with capacity for self-renewal and differentiation with a broad tissue distribution, and importantly, we observed a viability higher than 85% for all the MOF particles (Figure S20B). At this concentration of 0.2 mg/mL, cell morphology and adhesion were not affected after exposure to the MOF nanoparticles in both normal and cancer cells, which together with the high cell viability suggest good biocompatibility of these MOFs.

**Figure 3:**
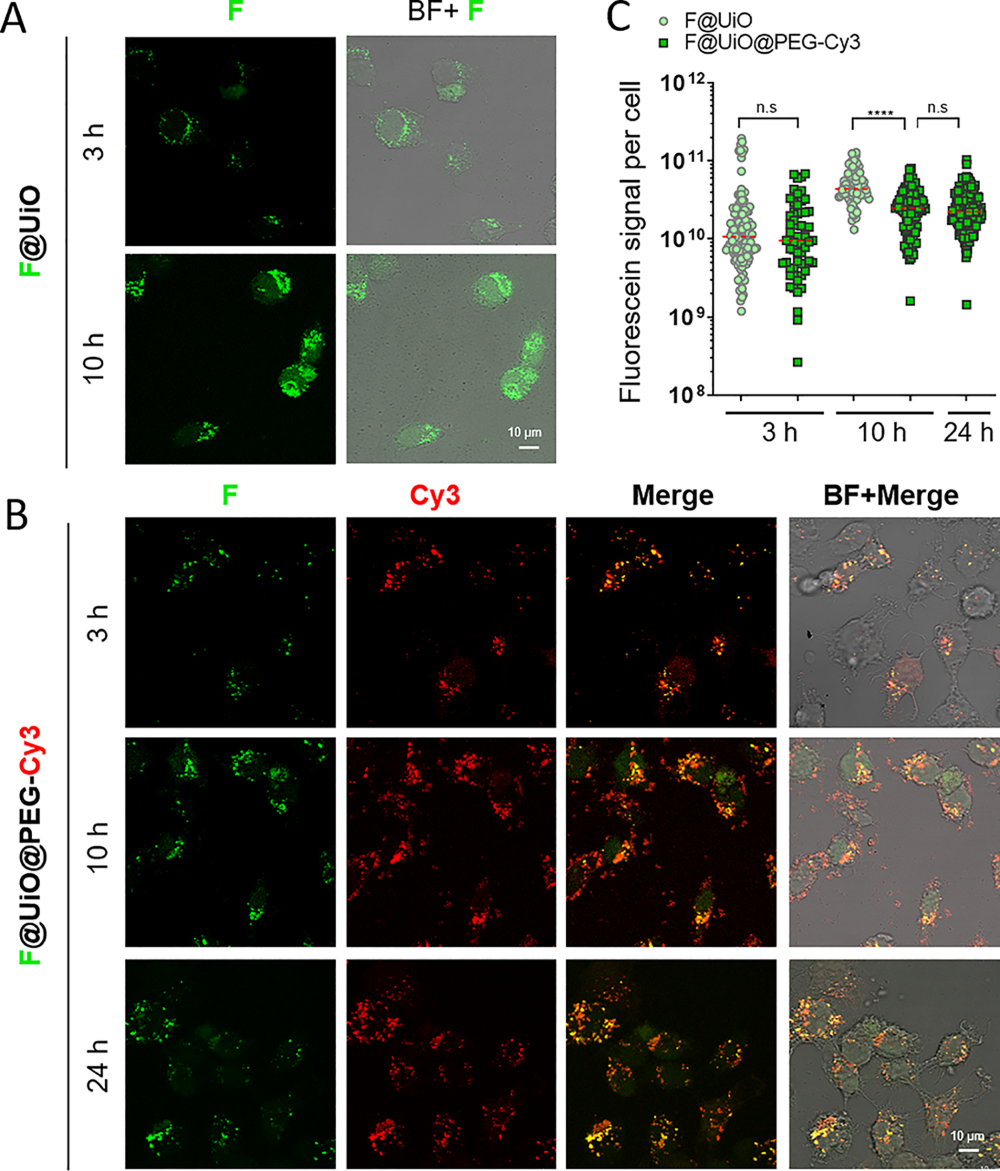
Cellular uptake of MOFs. Confocal microscopy images of HeLa cells incubated with (A) bare F@UiO and (B) functionalized F@UiO@PEG-Cy3 particles at equivalent MOF concentrations C_MOF_ of 0.2 mg/mL for 3, 10, or 24 h. C_MOF_ corresponds to the mg/mL of UiO, that is considering exclusively the UiO weight. Green (F) and red (Cy3) fluorescence channels, merge and bright field (BF) are shown; yellow marks indicate the colocalization between green and red channels. (C) Corresponding quantification of fluorescein signal per cell (*n* = 80 cells, logarithmic scale). Statistical analysis using a paired t-test was performed between the bare and functionalized particles (n.s: not statistically significant; ****p ≤ 0.0001).

The intracellular monitoring of the fluorescent MOF particles was first carried out at different exposure times (3, 10, and 24 h) by confocal microscopy. As shown in Figure 3, both the bare F@UiO and functionalized F@UiO@PEG-Cy3 particles were efficiently internalized with a similar rate at 3 h, as determined by quantifying the total fluorescein signal per cell. At 10 h the bare F@UiO displayed a 2-fold increase in cellular uptake as compared to the F@UiO@PEG-Cy3 particles (mean = 2.1 × 10^10^ versus 1.6 × 10^10^ a.u., respectively; Figure 3C), which is in line with previous studies showing similar reduced cellular uptake after PEGylation in the first hours.^10,33^ Note that no further differences in the uptake of F@UiO@PEG-Cy3 particles were found between 10 and 24 h, most likely because the particle exocytosis rate closely matched the endocytosis rate. This can be considered as a positive effect, since it suggests a low bioaccumulation of these MOFs. Similar results of the uptake kinetics of the MOF particles by HeLa cells were observed by flow cytometry (Figures S24–26). Although the intracellular fluorescence intensity showed slightly different initial kinetics for the endocytosis of the bare F@UiO and PEGylated F@UiO@PEG-Cy3 particles, after long incubation times (from 24 h) the total amount of internalized MOF particles was quite similar in both cases (Figure S26). This is consistent with previous findings reported by Fairen-Jimenez and co-workers^10^ for another Zr-MOF, specifically for the bare PCN-222 and its PEGylated derivative, which presented very similar cellular affinities at 24 h. On the contrary, PCN-128 showed great differences when comparing the cellular uptake of the bare and PEGylated particles, so the internalization of MOFs must be studied on a case-by-case basis. Control experiments with free fluorescein by confocal microscopy and flow cytometry (Figures S21 and S25) showed that this small hydrophilic molecule was not efficiently taken up or accumulated in cells at the concentration tested (i.e., equivalent amount to that present in the fluorescein-loaded MOF particles, see the ESI for details). This is not surprising, since the potential of MOFs as nanocarriers to internalize cargoes that cannot cross the cell membrane by themselves is widely recognized today. To further confirm that the MOF nanoparticles were internalized by cells and not merely attached onto the external surface, we took advantage of the optical sectioning capabilities of the laser scanning confocal microscope by collecting of z-stack images of cells treated with F@UiO@PEG-Cy3 particles for 3 h (Figure S22). The latter clearly showed that F@UiO@PEG-Cy3 particles were internalized and accumulated as clusters inside the cytoplasm of the cells.

On the other hand, a high degree of colocalization of both fluorescent channels in F@UiO@PEG-Cy3 particles (i.e., green from fluorescein loaded on MOF and red from PEG-Cy3 coating at the MOF surface) was observed not only at the first hours but even up to 10 h (Figure 3), indicating clearly that the particles retained their structural integrity during the uptake process, and also that the PEG coating was able to prevent undesirable premature cargo release. Control experiments with free PEG-Cy3 (Figures S21 and S25) demonstrated that such free ligands could not be transported into cells at the concentration used, so the potential detaching of the PEG surface coating from F@UiO@PEG-Cy3 particles prior to their endocytosis was ruled out. Furthermore, the finding that 89% of fluorescein signal was colocalized with the Cy3 signal also pointed out that the functionalized MOF particles maintained the integrity during their endocytosis and even after 24 h (Figure S23). This is the key feature for achieving a delayed and sustained release of the cargo over time as observed in the confocal images (Figure 3B), compared to the faster release of fluorescein molecules from the bare F@UiO particles, as shown by the green fluorescence not only punctuated in endosomes/lysosomes but also distributed throughout the cytoplasm (Figure 3A). This finding is consistent with the results presented in Figure 2D, where the amount of DOX released from DOX@UiO@PEG particles was minimal after 72 h of incubation in a buffer.

**Figure 4:**
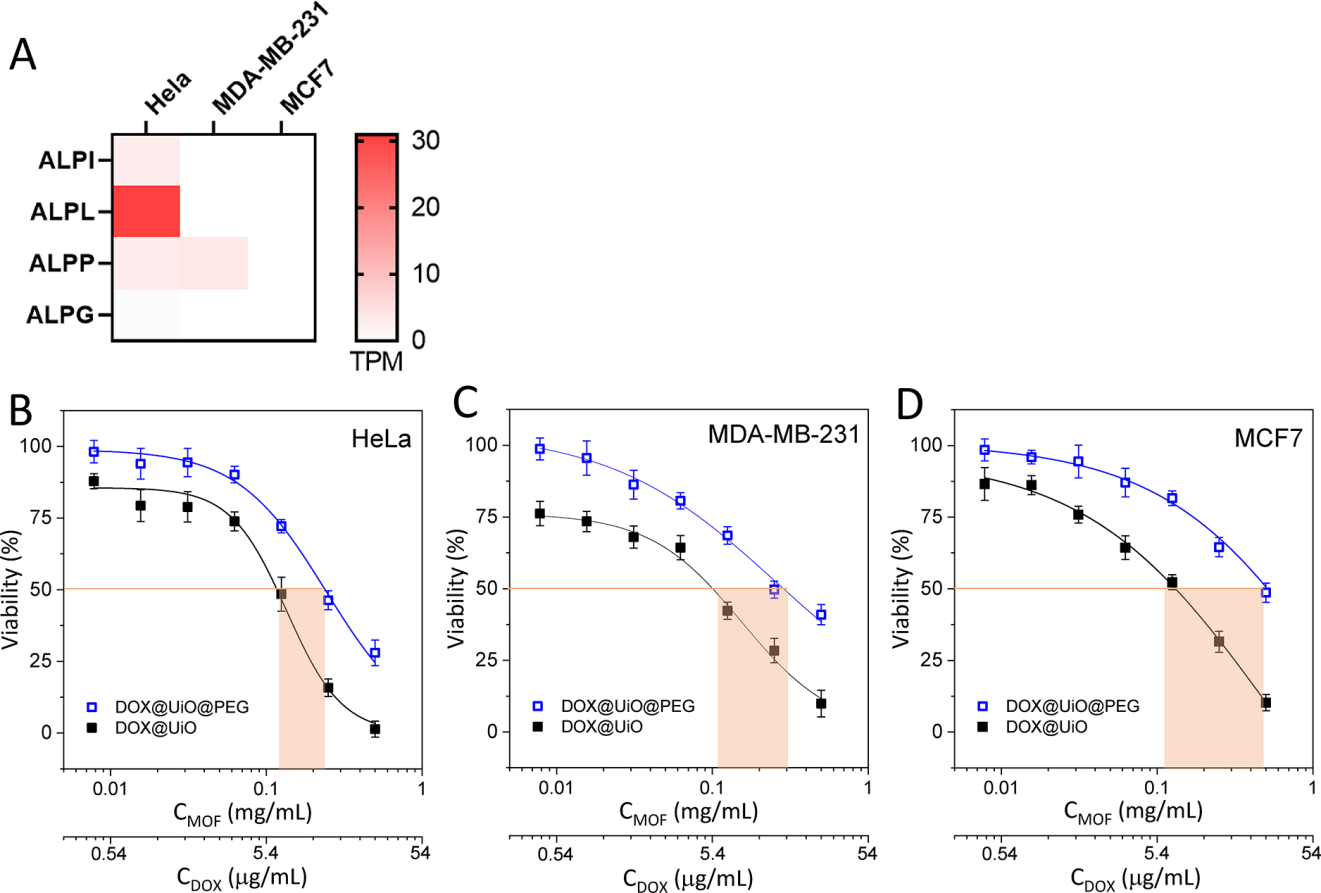
(A) RNA expression level of genes with phosphatase activity in three cell lines: HeLa, MDA-MB-231, and MCF7; data from CCLE and expression atlas EMBL-EBI. Cell viability (using CellTiter-Glo assay) of (B) HeLa, (C) MDA-MB-231, and (D) MCF7 under 72 h exposure to increasing concentrations of DOX@UiO and DOX@UiO@PEG particles. *C*_MOF_ corresponds to the mg/mL of UiO (considering exclusively the UiO weight). *C*_DOX_ corresponds to the concentration of DOX in μg/mL, knowing that the amount of DOX loaded in the MOF particles is 5.4 wt % as determined by UV–vis. Differences in the concentration of noncoated and PEG-coated particles needed to kill 50% of cells are marked with a pinkish rectangle.

Next, we sought to investigate whether the intracellular release of the cargo from the functionalized F@UiO@PEG-Cy3 particles (observed mainly from 24 h) was due to the potential PEG cleavage inside cells. To this end, we first carried out a deep quantitative analysis of confocal images of HeLa cells treated with F@UiO@PEG-Cy3 particles, observing that 72% of Cy3 signal was free (i.e., not colocalized with fluorescein signal) after 24 h (Figure S23). Interestingly, Hela cells express several isoforms of the alkaline phosphatase (ALPI, ALPL, and ALPP) as compared to MDA-MB-231 and MCF7 cells (Figure 4A). Of particular interest was the high expression levels of ALPL in Hela cells suggesting that these cells may be more efficient in cleaving the phosphate ester bond in F@UiO@PEG-Cy3 particles by ALP enzymolysis. To further investigate and exploit this hypothesis within the context of controlled drug delivery, we performed studies with doxorubicin-loaded MOF particles with and without the PEG coating (DOX@UiO and DOX@UiO@PEG) on the three aforementioned cell lines (Figure 4B–D). In all cell lines, the PEG coating presented a protective effect on the encapsulated DOX, resulting in a significant lower toxicity of DOX@UiO@PEG compared to DOX@UiO particles, by assuming that at the long incubation time of 72 h both particles were internalized to a comparable extent as suggested by the flow cytometry data. Most importantly, the cell line with the highest expression of genes with APL activity (HeLa; Figure 4B) showed the least protection, consistent with their ability to cleave the PEG from the MOF surface, and then allowing a faster drug release. In contrast, MCF7 cells that lack expression of ALP isoforms had a four-fold increase in the particle concentration needed to kill 50% of cells (Figure 4D), as a result of the sustained DOX release from the PEGylated MOF due to the good intracellular stability of DOX@UiO@PEG in the absence of ALP activity. Although it is likely that any cell capable of endocytosing these MOFs could eventually degrade them and release the cargo, the results showed a significant preferential release of DOX in cells with enhanced ALP activity. These findings clearly confirmed the enzyme-responsive release mechanism for the DOX-loaded MOF system as designed, which could be further modified with active targeting ligands for specific retention and uptake by diseased cells, and thereby expanding the potential of the system for targeted therapy.

## Conclusions

We have demonstrated for the first time the intracellular enzyme-triggered controlled-drug release capability of nanosized UiO-66 functionalized with N_3_–PEG–PO_3_ ligands, exploiting the successful cleavage of the phosphate ester bond between the PEG ligand and the MOF surface by the ALP activity. We evidence that the here synthetized N_3_–PEG–PO_3_ ligands played three key functions: (1) to increase the colloidal stability of nanosized MOFs in phosphate-containing media, (2) to endow the MOF nanoparticles with “clickable” potential for the attachment of additional functionalities (e.g., imaging probes), and (3) to provide ALP-responsiveness for controlling the release kinetics of the cargo as a function of the ALP activity in targeted cells. It is noteworthy that ALP is encoded by four independent genes for which the expression may be tissue specific; however, ALP is overexpressed on some cancer cells (mainly pancreatic, prostate, colon, lung, and gastric cancers) and other pathologies (such as liver and osteoblast dysfunctions) and is thus considered an important clinical marker. Despite this fact, its potential to act as an endogenous stimulus to trigger the release of a drug for targeted chemotherapy is underexploited and warrants further investigation. Altogether, this work provides incentive for designing novel enzyme-responsive MOF-based nanoplatforms for targeted controlled drug delivery and other biomedical applications.

## Supporting Information Available

Full experimental procedures, characterization details, and additional supporting data and figures (PDF)
