# Supplementary material for: Enzyme-Responsive Zr-Based Metal–Organic Frameworks for Controlled Drug Delivery: Taking Advantage of Clickable PEG-Phosphate Ligands
Source: ACS Appl Mater Interfaces. 2023 May 30;15(23):27600–11. doi: 10.1021/acsami.3c03230 (PMC10273175; doi:10.1021/acsami.3c03230)
Supplement: Supplementary file 1 — am3c03230_si_001.pdf [file am3c03230_si_001.pdf]

# SUPPORTING INFORMATION

# Enzyme-responsive Zr-based Metal-Organic Frameworks for Controlled Drug Delivery: Taking Advantage of Clickable PEG-Phosphate Ligands

Carolina Carrillo-Carrión,*[a] Valentine Comaills,[b] Ana M. Visiga,[a] Benoit R. Gauthier[b,c] and Noureddine Khiar*[a]

[a] Institute for Chemical Research (IIQ), CSIC-University of Seville, 41092 Sevilla, Spain. E-mail: carolina.carrillo@csic.es; khiar@iiq.csic.es

[b] Andalusian Center for Molecular Biology and Regenerative Medicine (CABIMER), Junta de Andalucía-University of Pablo de Olavide-University of Seville-CSIC, 41092 Sevilla, Spain.

[c] Centro de Investigación Biomédica en Red de Diabetes y Enfermedades Metabólicas Asociadas (CIBERDEM), 28029 Madrid, Spain

| TABLE OF CONTENTS | Page |
|---|---|
| **S1. General experimental remarks** | 2 |
| **S2. Synthesis and characterization of PEG-Phosphate ligands** | 5 |
| **S3. Synthesis and functionalization of MOF nanoparticles** | 9 |
| **S4. Morphological/structural and compositional characterization of MOFs** | 10 |
| **S5. Doxorubicin loading and characterization** | 16 |
| **S6. Enzyme-triggered release studies** | 18 |
| **S7. Click chemistry on MOF nanoparticles** | 20 |
| **S8. Cell studies** | 23 |
| **S9. References** | 26 |

## S1. General experimental remarks

**Chemicals:** All reagents were obtained from commercial sources and were used without further purification unless otherwise stated.

**Nuclear Magnetic Resonance Spectroscopy (NMR)**: $^{1}H$ NMR and $^{31}P$ NMR spectra were recorded on a 400 MHz Bruker Avance III HD spectrometer and referenced to residual solvent peaks.

**Electrospray Ionisation Mass Spectrometry (ESI-MS)**: Mass spectra were carried out with a high-resolution Q-Exactive hybrid quadrupole orbitrap mass spectrometer from Thermo Scientific.

**Transition Electron Microscopy (TEM):** TEM images were acquired using a JEOL 2100Plus operated at 200 kV. Samples were prepared by drying, under ambient conditions, a diluted dispersion of the particles on 200 mesh copper grids coated with Formvar/carbon film. Elemental composition was analysed by Energy Dispersive X-Ray Analysis (EDX) using the same TEM instrument with an integrated Oxford INCA EDX system.

**Scanning Electron Microscopy (SEM):** SEM images were acquired using a with a HITACHI S4800 field emission microscope operating at 2 kV in secondary electron and backscattered electron modes. Samples were prepared by drying, under ambient conditions, a diluted dispersion of the particles on a silicon wafer substrate.

**Dynamic Light Scattering (DLS) and Zeta-Potential (ζ-potential):** Measurements were performed using a Malvern Zetasizer Nano ZSP equipped with a 10 mW He–Ne laser operating at a wavelength of 633 nm and fixed scattering angle of 173˚. For DLS analysis, diluted samples were loaded into a quartz cuvette and then three measurements, each consisting of twelve data runs, were taken at room temperature (25°C) after an equilibration step of 120 sec. The zeta-potential of samples in aqueous solutions (water or buffer solutions) were measured with laser Doppler anemometry (LDA) by using the same Malvern Zetasizer Nano ZSP instrument.

**Powder X-Ray Diffraction (PXRD):** X-ray analysis of the crystalline powder of samples was performed using a Bruker D8-Advance Diffractometer. X-ray radiation of Cu Kα was used and the measurement range was 5°–70° (2θ) with a step of 0.02°.

**Fourier-Transform Infrared Spectroscopy (FTIR)**: FTIR analysis was performed on a Bruker Tensor 27 spectrophotometer; spectra were recorded at room temperature in the range 400–4000 $cm^{-1}$ with a resolution of 4 $cm^{-1}$. Dried samples were prepared on a KBr pellet under a pressure of 0.01 torr.

**$N_2$ physisorption analysis**: $N_2$ sorption isotherms were carried out at 77 K on a Quantachrome Autosorb-iQ-2 MP/XR gas sorption analyser. Samples were degassed under vacuum at 120 ºC for 24 h. The Quantachrome ASiQwin operating software was used for analysis.

**Thermogravimetric analysis (TGA)**: TGA of powder samples was performed using a Thermal Advantage SDT-600 instrument with a general heating profile from 30 to 700 °C and using a heating rate of 5 °C·min-1 under air.

**Inductively Coupled Plasma-Optical Emission Spectroscopy (ICP-OES):** ICP-OES was performed using a ThermoFisher Scientific iCAP 7200 ICP-OES Duo. Samples were prepared by microwave-assisted digestion with aqua regia (3:1 nitric acid to hydrochloric acid). ICP-OES was used to determine the amount of Zr and P in the particles. Zr and P elemental certified standards were used to prepare the calibration curves.

**UV-Vis Spectroscopy**: Spectra were recorded using a Perkin Elmer Lambda 750. 1 cm quartz optical cells were utilized to make measurements.

**High-performance liquid chromatography coupled with mass spectrometry (HPLC-MS):** HPLC-MS analyses were done using a Waters Alliance 2695 HPLC coupled to a ESI-ion trap mass spectrometer instrument (Bruker AmaZon) equipped with Compass HyStar software.

**Cell Culture**: HeLa, MDA-MB-231 and MCF7 cell lines were maintained at 37 °C and 5% $CO_2$ in DMEM (Dulbecco's Modified Eagle Medium) supplemented with 10% (v/v) fetal bovine serum, 100 units/mL penicillin, and 100 µg/mL streptomycin. The supplemented medium is then named as complete medium. Normal Mesenchymal Stem Cells (MSC; ATCC PCS-500-011) were grown as previously reported.[1] 5,000 cells were seeded in completed media in 96 well plates (opaque Tissue Culture treated plate, Pierce) for 24 h prior to the addition of the MOF nanoparticles.

**Viability Assay**: The concentration-dependent viability of the different prepared MOF nanoparticles was investigated using the CellTiter-Glo Proliferation Assay (Promega). Briefly, cells were seeded on a 96-well plate at a density of 5,000 cells/well for 24 h. Prior to each experiment, suspensions of MOF nanoparticles (stock solution at 20 mg/mL in methanol) were freshly diluted in complete cell medium at desired concentrations. Cell viabilities of MOF nanoparticles were determined after an incubation period of 24 h at 37 ºC, 5% $CO_2$. Additionally, cell viability after exposure to DOX-loaded MOF nanoparticles and free DOX was assayed after 72 h of incubation time. Standard procedure of CellTiter-Glo assay was performed. Luminescence signals were normalized to corresponding untreated controls. Luminescence was read with a Varioskan Lux Reader (Thermo Fisher).

**Confocal Microscopy**: 20,000 cells were grown on 15 mm coverslips coated with poly-L-lysine within a 12-well plate and incubated overnight in complete medium at 37 °C, 5% $CO_2$. Cells were then treated with 0.2 mg/mL of MOF nanoparticles (or with the equivalent amounts of free fluorescein and free PEG-Cy3) diluted in media for 3 h, 10 h, or 24 h incubation. Cells without addition of MOF nanoparticles were used as controls. At the end of the incubation time, cells were washed 3 times with PBS and then fixed with 4% paraformaldehyde for 15 min at room temperature. Following fixation, cells were washed 2 times with PBS and then coverslips were mounted on microscope slides using fluorescent mounting medium (Dako). Alternatively, cells were also incubated with DAPI (1µg/mL) for 20 min before mounting. Confocal imaging was carried out using a Leica TCS SP5 laser scanning confocal microscope and using either objective 40x or 60x. from the CABIMER microscopy core facility. Each region was picture with an average of 30 Z stacks of 0.28 and 0.2 micrometer distance. Bright field, DAPI, Cy3 and GFP filters were used to detect the Fluorescein and Cy3 fluorescent molecules as well as the DNA from DAPI stain. Colocalization values were calculated using the microscopy image analysis software IMARIS (Oxford instruments). Total fluorescence intensity per cell was calculated using the Image J software by doing a Z-projection and SUM slide, and corrected by subtracting signal from the background. Statistics were performed with the Graphpad PRISM 9 software.

**Alkaline expression in cell lines**: RNA expression level of the different isozymes of alkaline phosphatase was observed using data from the cancer cell line encyclopedia CCLE and expression was observed using the website from the atlas EMBL-EBI. Cell lines with different expression level were used, specifically HeLa, MDA-MB-231 and MCF7. *TPM* (protein-coding transcripts per million).

**Flow Cytometry**: 100,000 HeLa cells were treated with the MOF nanoparticles at 0.2 mg/mL or with the equivalent amounts of free fluorescein and free PEG-Cy3 (as present in MOF particles) for 3, 10 and 24h before being analysed by flow cytometry (CABIMER Cytometry Core facility). Single cells suspension was analysed using the LSRFortessa X-20 with the Ds-RED and A488 filters. >10000 events for each condition were collected. Figures and quantifications were obtained with the FlowJo software.

## S2. Synthesis and characterization of PEG-Phosphate ligands

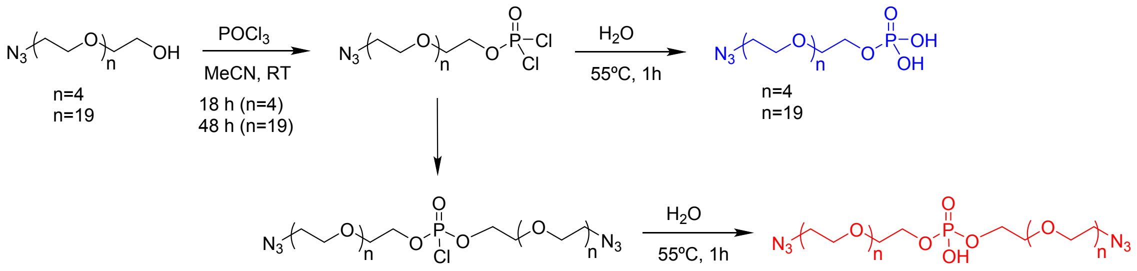


**Scheme S1**. Synthesis of target ligands (blue) and potential formation of an undesirable subproduct (red).

**Synthesis of $N_3$-(PEG)$_5$-$PO_3$.** Phosphorus oxychloride ($POCl_3$; 1 g, 6.65 mmol) was added to 10 mL of dry acetonitrile (MeCN) placed in a flame-dried two-neck flask and under argon atmosphere, followed by the dropwise addition over 15 min of 14-azido-3,6,9,12-tetraoxatetradecan-1-ol ($N_3$-PEG(5)-OH; 0.35 g, 1.33 mmol) in 10 mL of dry MeCN. The resulting solution was stirred at room temperature for 18 h. After that, the solvent was evaporated under reduced pressure vacuum conditions, and 40 mL of water was added dropwise over 30 min. The mixture was reacted for 1 h at 55 ºC, and finally the solvent was removed under vacuum. Purification was carried out by chromatographic column using as eluent a mixture of DCM/MeOH/$H_2O$ (7:2.5:0.5) to give $N_3$-(PEG)$_5$-$PO_3$ as a yellow sirup with a yield of 94 %.

**$^1$H NMR (400 MHz, $D_2O$)**, δ: 3.97 (dd, $J$ = 9.1, 6.1 Hz, 1H), 3.69 (d, $J$ = 3.4 Hz, 8H), 3.55 – 3.39 (m, 1H). **$^{31}$P RMN (400 MHz, $D_2O$)**, δ: 0.679. **ESI-MS**, calcd. for C10H22O8N3NaP $[M+Na]^+$=366.1037, found 366.1034.

**Synthesis of $N_3$-(PEG)$_{20}$-$PO_3$.** Phosphorus oxychloride ($POCl_3$; 1.3 g, 8.11 mmol) was added to 30 mL of dry MeCN in a flame-dried two-neck flask under argon atmosphere, followed by the dropwise addition over 30 min of alpha-azido-omega-hydroxy icosa(ethylene glycol) ($N_3$-PEG(20)-OH; 1.5 g, 1.62 mmol) in 20 mL of dry MeCN. The resulting solution was stirred at room temperature for 48 h, and afterwards the solvent was evaporated under vacuum. 40 mL of water was added dropwise over 30 min. The mixture was reacted for 1 h at 55 ºC, and finally the solvent was removed under vacuum. Purification was carried out by chromatographic column using as eluent a mixture of DCM/MeOH/$H_2O$ (7:2.5:0.5) to give $N_3$-(PEG)$_{20}$-$PO_3$ as a yellow sirup with a yield of 83 %.

**$^1$H NMR (400 MHz, $D_2O$)**, δ: 3.99 (dd, $J$ = 9.2, 6.2 Hz, 1H), 3.66 (d, $J$ = 20.6 Hz, 38H), 3.56 – 3.45 (m, 2H). **$^{31}$P RMN (400 MHz, $D_2O$)**, δ: 0.394. **ESI-MS**, calcd. for C40H82O23N3NaP $[M+Na]^+$=1026.4969, found 1026.4959.

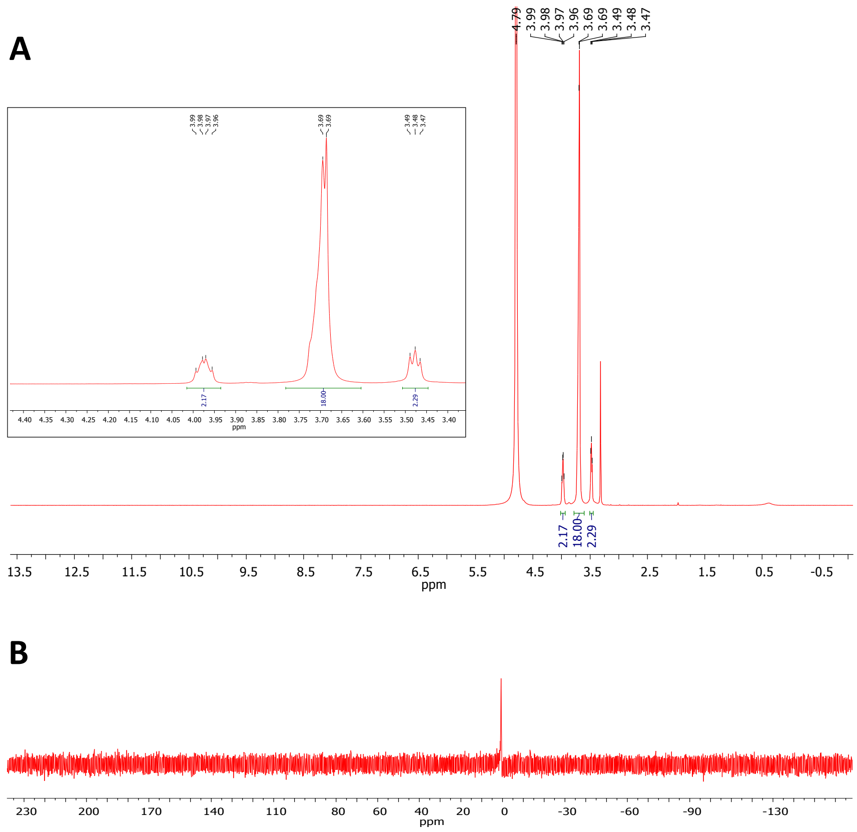


**Figure S1**. (A) $^{1}H$ NMR spectrum and (B) $^{31}P$ NMR spectrum of $N_3$-(PEG)$_5$-$PO_3$.

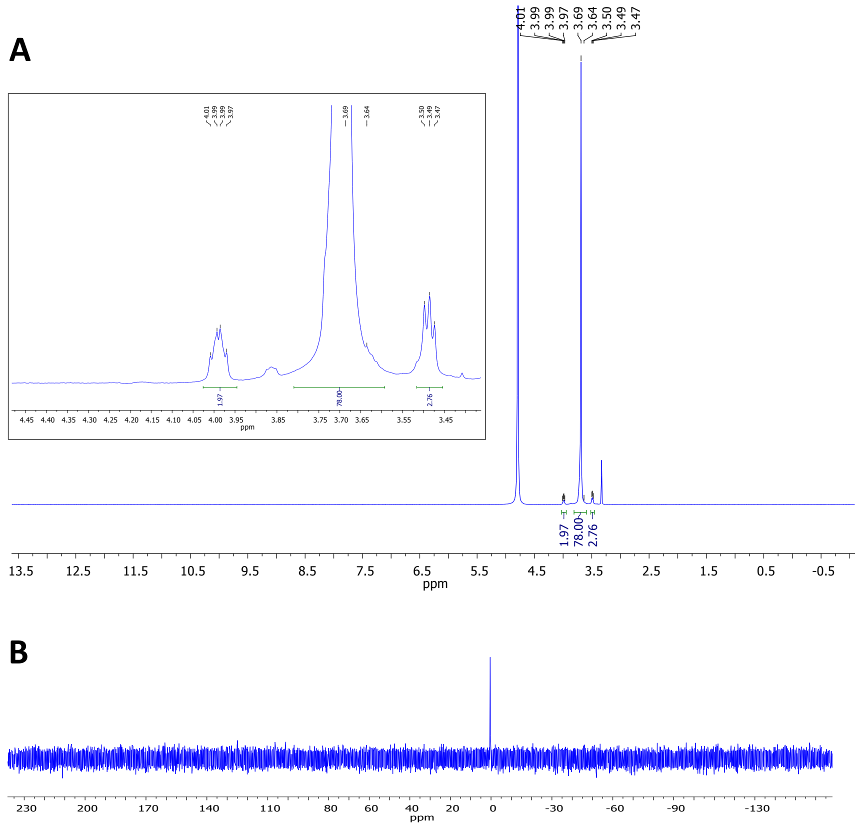


**Figure S2**. (A) $^{1}H$ NMR spectrum and (B) $^{31}P$ NMR spectrum of $N_3$-(PEG)$_{20}$-$PO_3$.

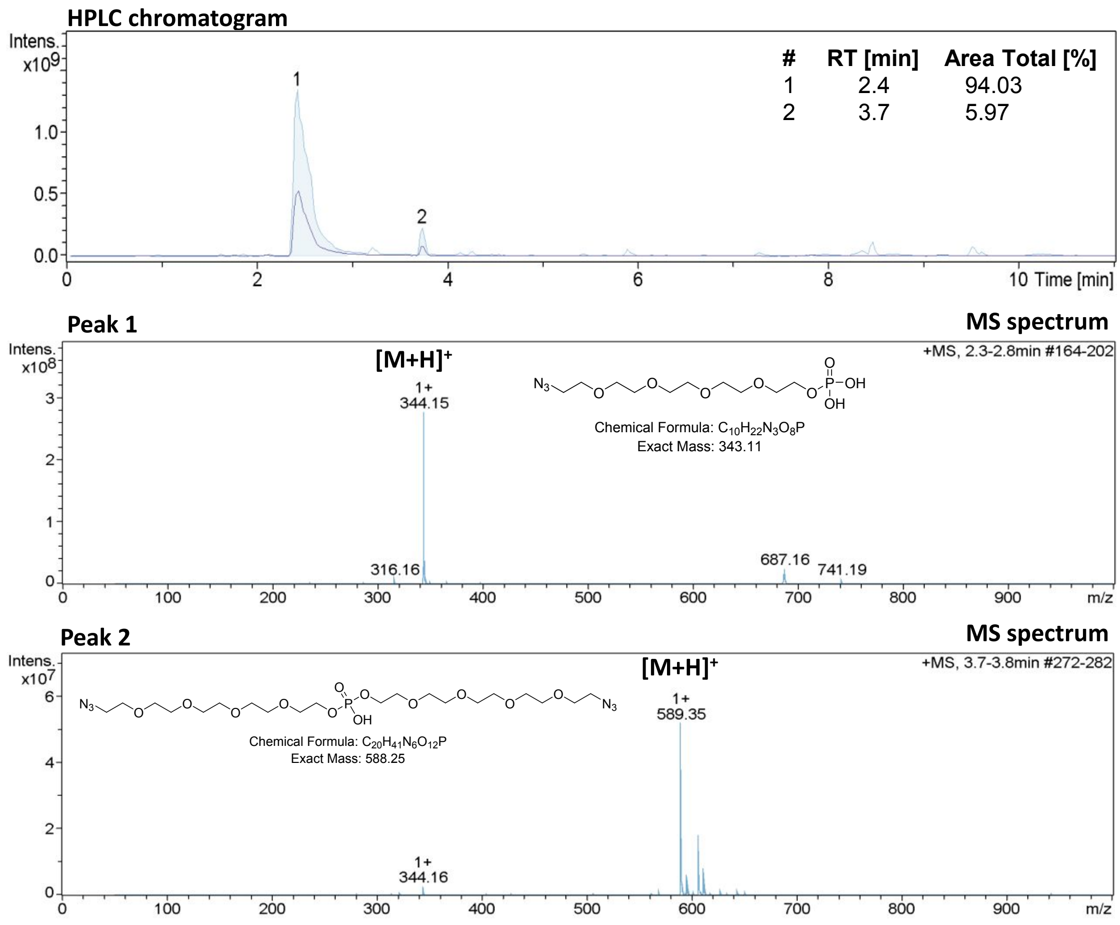


**Figure S3**. HPLC-MS analysis of the crude mixture obtained in the synthesis of $N_3$-(PEG)$_5$-$PO_3$, showing the MS spectra of the target compound (peak 1) and the side product (peak 2).

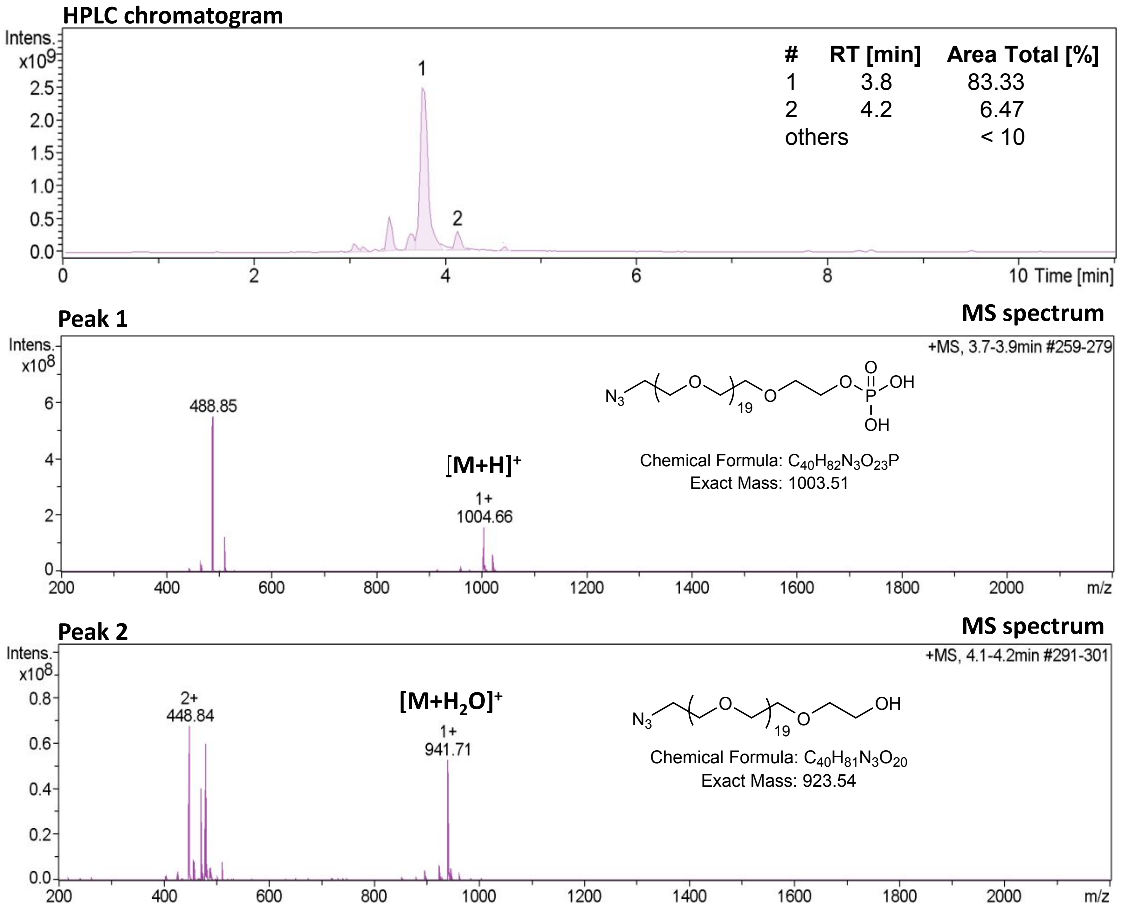


**Figure S4**. HPLC-MS analysis of the crude mixture obtained in the synthesis of $N_3$-$(PEG)_{20}$-$PO_3$, showing the MS spectra of the target compound (peak 1) and one identified side product (peak 2).

## S3. Synthesis and functionalization of MOF nanoparticles

**Synthesis of UiO-66 nanoparticles (UiO).** 1,4-benzenedicarboxylic acid ($H_2BDC$; 500 mg, 3 mmol) and $ZrCl_4$ (156 mg, 0.67 mmol) were dissolved in 40 mL DMF using an ultrasonic bath (~20 min), followed by addition of acetic acid (20 mL). The mixture was heated at 100 ºC for 90 min (optimized growth time). After cooling to room temperature, the obtained particles were collected by centrifugation (13,000 rpm, 5 min), followed by washing twice with fresh DMF and 3 times more with MeOH. The final purified UiO nanoparticles were redispersed in MeOH at a concentration of 10 mg/mL and stored in the fridge until use.

**Surface functionalization of UiO with PEG-Phosphate ligands.** The ligand/MOF ratio and solvent were optimized individually for both PEG ligands, $N_3$–$(PEG)_n$–$PO_3$ (n=5 and 20), determining the functionalization efficiency and the amount of PEG ligand bound to the MOF particles in each case. The functionalization efficiency was calculated by quantifying the amount of non-bound PEG ligand (remaining in the supernatant after purification of the particles) by HPLC and applying the following equation:

$$\%\ \text{Efficiency} = \frac{\mu mol_{\text{PEG bound}}}{\mu mol_{\text{PEG added}}} \times 100 = \frac{\mu mol_{\text{PEG added}} - \mu mol_{\text{PEG non-bound}}}{\mu mol_{\text{PEG added}}} \times 100$$

According to results (Table S1), the same experimental conditions were selected as the optimal regardless of the PEG length. Note that the use of large excess of PEG ligands led only to slight increases in the amount of PEG bound to the MOF (i.e., wt% PEG), and therefore it is not justified from a cost-effective point of view.

The final optimized functionalization procedure is the following. A methanolic suspension of UiO particles (1 mL, at 10 mg/mL) was placed in a glass vial and then an aqueous solution of $N_3$–$(PEG)_n$–$PO_3$ ligands (1 mL, at 10 mM) was added under gentle stirring. The mixture was stirred overnight (ca. 18 h) at room temperature, and the next day the MOF nanoparticles were centrifuged to remove the unreacted PEG ligands, and washed with Milli-Q water 3 times, redispersed in either water or methanol at a concentration of 10 mg/mL and stored in the fridge until use.

**Table S1**. Optimization data for the functionalization of UiO nanoparticles with $N_3$-$(PEG)_n$-$PO_3$ ligands (n = 5 and 20). The selected optimal conditions are shaded.

| $N_3$-$(PEG)_n$-$PO_3$ | PEG/MOF ratio added (µmol/mg) | Solvent | Efficiency (%) | PEG (wt%)[a] | PEG (wt%)[b] |
|---|---|---|---|---|---|
| n=5 | 1 | MeOH:$H_2O$ (1:1) | 87 | 23.0 | 19.0 |
| n=5 | 2 | MeOH:$H_2O$ (1:1) | 53 | 26.7 | |
| n=5 | 4 | MeOH:$H_2O$ (1:1) | 27 | 27.0 | |
| n=5 | 1 | MeOH | 64 | 18.0 | |
| n=20 | 1 | MeOH:$H_2O$ (1:1) | 74 | 42.6 | 38.4 |
| n=20 | 2 | MeOH:$H_2O$ (1:1) | 42 | 45.7 | |
| n=20 | 4 | MeOH:$H_2O$ (1:1) | 22 | 46.9 | |
| n=20 | 1 | MeOH | 39 | 28.1 | |

[a] determined by HPLC
[b] determined by ICP-OES

## S4. Morphological/structural and compositional characterization of MOFs

The morphology (shape and size) and the homogeneity of the as-prepared UiO and UiO@$PO_3$–$(PEG)_{20}$–$N_3$ particles were investigated with SEM and TEM. Representative TEM images the MOF particles prepared under optimized conditions are shown in Figure S5. The reaction time (crystal growth time) of the UiO nanoparticles was found to influence significantly not only the size of the particles, but also their morphology. Figure S6 shows the evolution of the morphology of the UiO nanoparticles over time, and according to that, 90 min was selected as the optimal time.

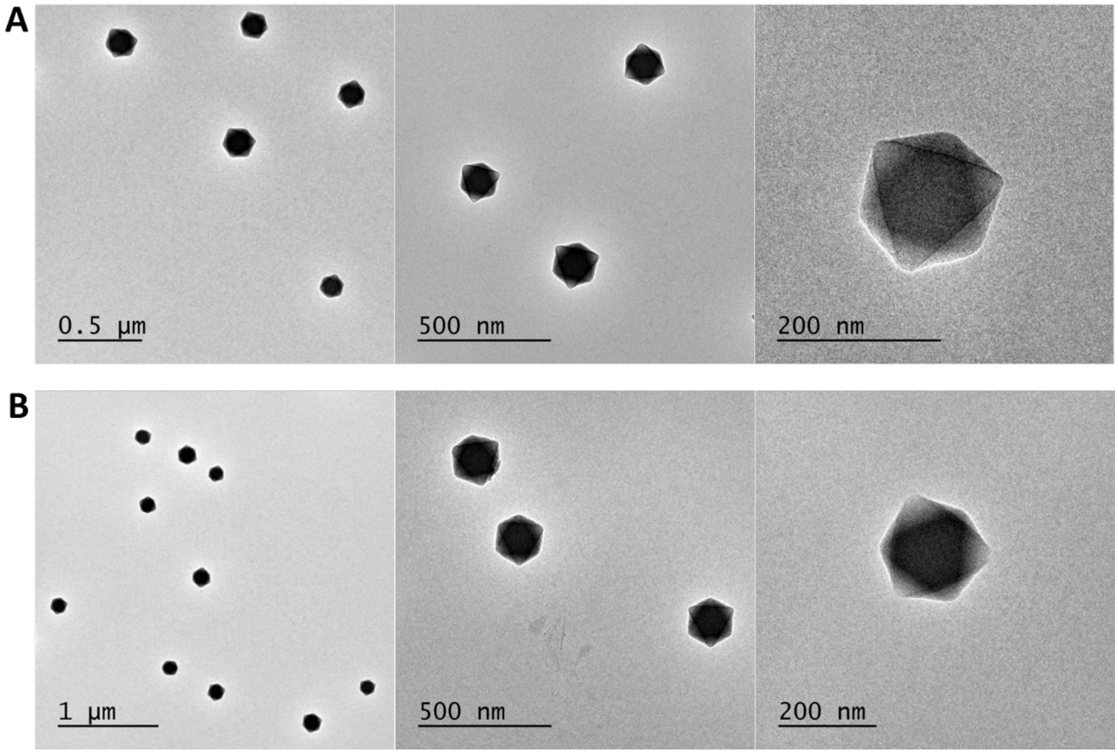


**Figure S5**. Representative TEM images of (A) UiO and (B) UiO@$PO_3$–$(PEG)_{20}$–$N_3$ particles.

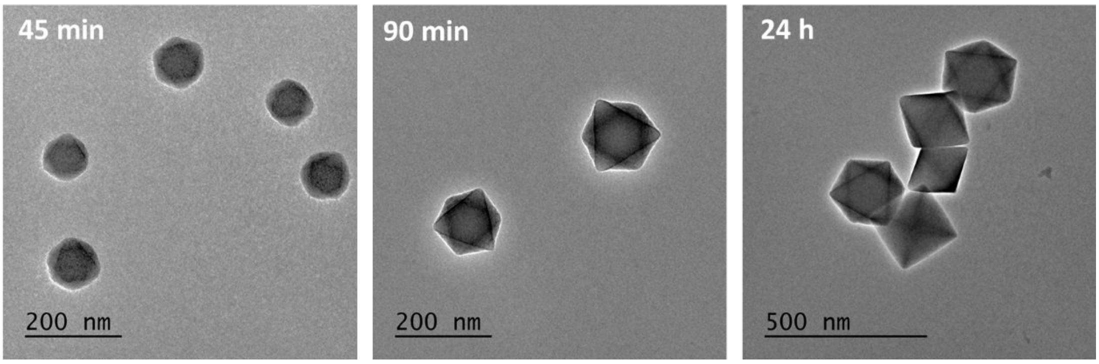


**Figure S6**. TEM images of UiO nanoparticles obtained after different reaction times, 45 min, 90 min, and 24 h.

ICP-OES analyses of the UiO nanoparticles after their functionalization with $N_3$–$(PEG)_n$–$PO_3$ ligands (for n = 5 and 20) was used to estimate the amount of PEG incorporated in each case (Table S2). The calculations were performed using $Zr_6O_4(OH)_4(BDC)_6$ as the empirical formula for UiO-66, that is, considering an idealized defect-free structure for UiO-66.

**Table S2**. ICP-OES analyses of UiO@$PO_3$–$(PEG)_n$–$N_3$ particles.

| Sample | P (mg/L) | Zr (mg/L) | PEG loading (wt%) |
|---|---|---|---|
| UiO@$PO_3$–$(PEG)_5$–$N_3$ | 2.65 | 41.20 | 19.0 |
| UiO@$PO_3$–$(PEG)_{20}$–$N_3$ | 2.49 | 42.57 | 38.4 |

TGA and DSC curves of the UiO and UiO@$PO_3$–$(PEG)_{20}$–$N_3$ particles under air are shown in Figure S7, showing the thermal stability of the UiO nanoparticles before and after their functionalization. Comparison of these curves allowed to determine the amount of PEG incorporated within the UiO particles.

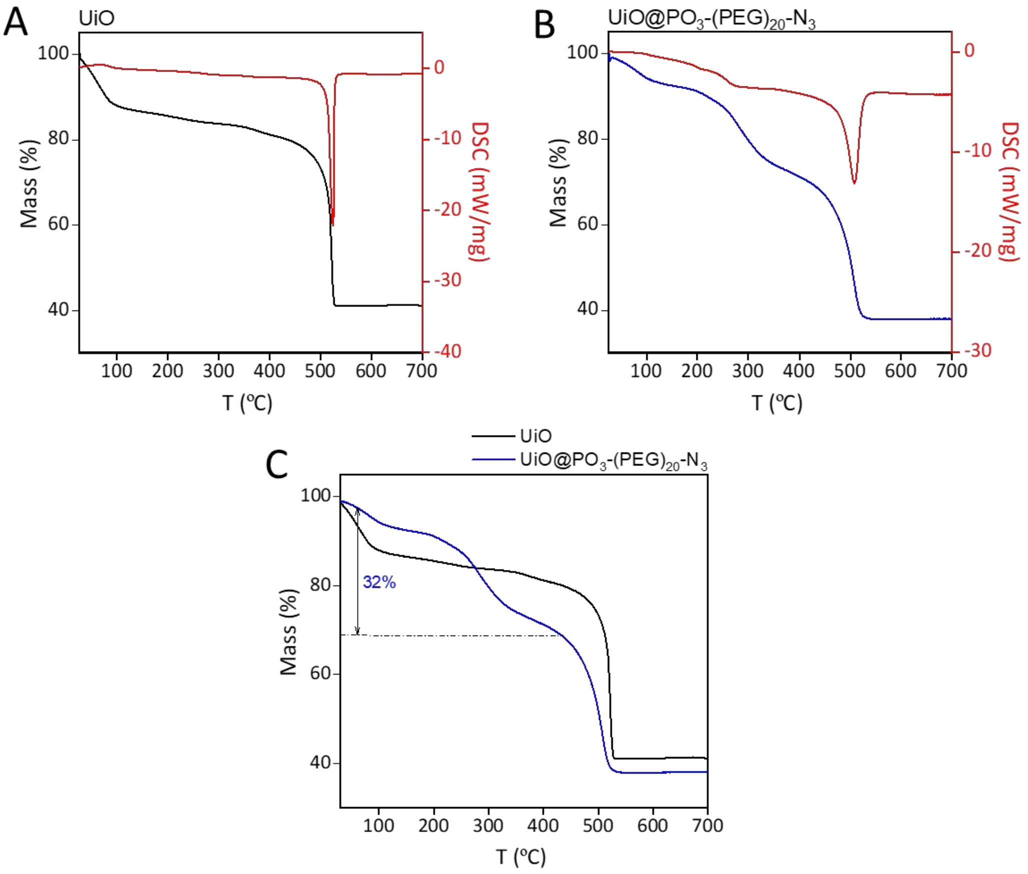


**Figure S7**. TGA and DSC profiles under air of (A) UiO and (B) UiO@$PO_3$–$(PEG)_{20}$–$N_3$ particles. (C) Comparison of TGA curves before and after the functionalization, indicating the estimated amount of PEG in the UiO@$PO_3$–$(PEG)_{20}$–$N_3$ particles.

As expected, the pristine UiO-66 had a distinct endothermic peak above 500 °C, consistent with the reported temperature of decomposition of UiO-66 and the formation of inorganic $ZrO_2$ as residual product under aerobic conditions. There was a first mass loss step before 100 °C, which is attributed to ambient moisture potentially adsorbed since the sample was not stored in vacuo or under dry conditions. The second gradual mass loss between 200–400°C is characteristic for the UiO family MOFs and is associated to the dehydration of the zirconium oxoclusters, $[Zr_6O_4(OH)_4L_6]$ (where L=ligand) and adopting then the formation $[Zr_6O_6L_6]$.[2] As for the UiO@$PO_3$–$(PEG)_{20}$–$N_3$ particles, there is also a sharp weight loss from 450 °C to 550 °C, which is belonged to the decomposition of UiO-66, demonstrating that the thermal stability of the MOF structure remained almost unchanged in spite of the functionalization with the $N_3$–$(PEG)_{20}$–$PO_3$ ligands. The first weight loss of ca. 32 wt% in UiO@$PO_3$–$(PEG)_{20}$–$N_3$ particles is associated with the degradation of the PEG ligands attached to the MOF surface, and quantified as around 32 wt%.

In order to have more information of the composition of the functionalized particles, we performed TEM-EDX analyses, observing some differences in the elemental composition found in the pristine UiO particles and after their functionalization. As shown in Figure S8, UiO particles had signals corresponding to Zr and O elements, while N and P (coming from the ligands) seemed to be only presence in the functionalized particles. Nevertheless, it must be noted that the signals corresponding to Zr and P elements cannot be properly separated by EDX spectroscopy, since the Lα line for Zr is 2.042 keV and the Kα line for P is 2.012 keV, and therefore this technique does not lead to a reliable identification of the elements or a precise quantification of the elemental composition in the particles.

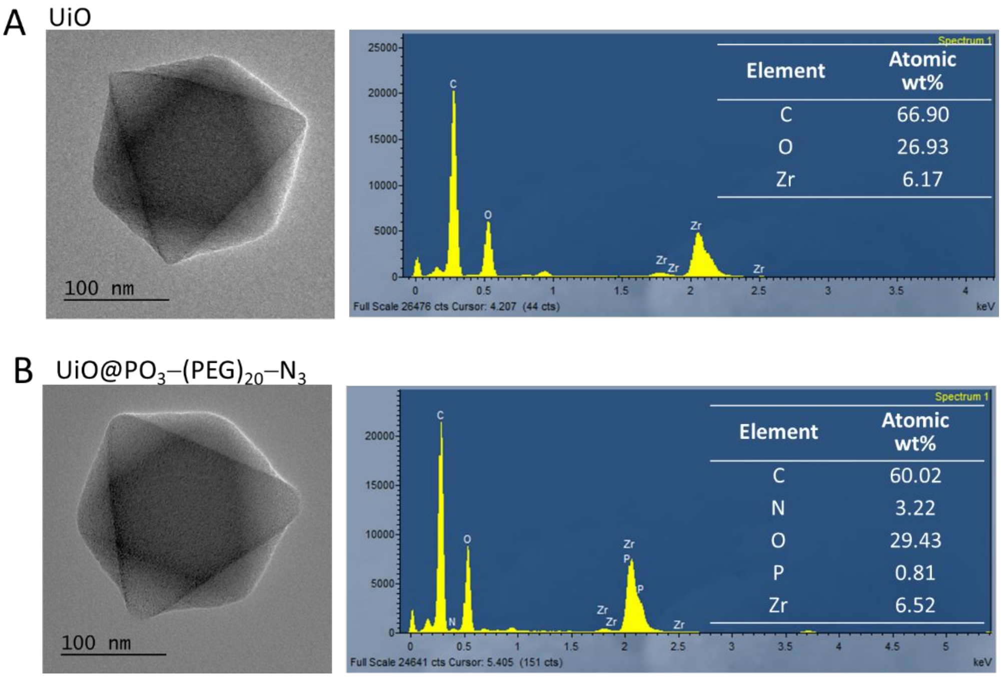


**Figure S8**. TEM-EDX analyses of the (A) UiO and (B) UiO@$PO_3$–$(PEG)_{20}$–$N_3$ particles. TEM images, the accumulative EDX spectra of a single particle, together with the estimated atomic concentration percentages of elements are shown.

DLS and LDA measurements were used to investigate the changes in the hydrodynamic sizes and surface charge of the UiO nanoparticles after their functionalization with the $N_3$–(PEG)$_n$–$PO_3$ (n=5 and 20) ligands (Table S3), as well as to study their colloidal stability over time in PBS (0.1 M, pH=7.4). DLS measurements were performed at different time points (Table S4). The results clearly showed clearly that only the long PEG ligand, $N_3$–(PEG)$_{20}$–$PO_3$, was able to protect UiO nanoparticles from the attack of phosphate ions in the long term.

**Table S3**. Hydrodynamic size (mean value ± SD), polydispersity index (PDI), and ζ-potential (mean value ± SD) of UiO, UiO@$PO_3$–(PEG)$_5$–$N_3$ and UiO@$PO_3$–(PEG)$_{20}$–$N_3$ particles as dispersed in Milli-Q water. SD values correspond to the standard deviation of the size mean value as obtained from several repetitions (n=3) of the measurement.

| Sample | Size (nm) | PDI | ζ-potential (mV) |
|---|---|---|---|
| UiO | 198 ± 4 | 0.024 | -14.8 ± 0.8 |
| UiO@$PO_3$–(PEG)$_5$–$N_3$ | 215 ± 3 | 0.026 | -20.7 ± 1.0 |
| UiO@$PO_3$–(PEG)$_{20}$–$N_3$ | 224 ± 5 | 0.083 | -22.0 ± 1.2 |

**Table S4**. Hydrodynamic sizes (mean value ± SD) as derived from DLS measurements over time of UiO, UiO@$PO_3$–(PEG)$_5$–$N_3$ and UiO@$PO_3$–(PEG)$_{20}$–$N_3$ particles dispersed in PSB (0.1 M, pH=7.4). Under some conditions, the nanoparticles were dissolved and could not be analysed (n.a.) Data correspond to raw data depicted in Figure 1F.

| | Size (nm) | | |
|---|---|---|---|
| **Time (h)** | **UiO** | **UiO@$PO_3$–(PEG)$_5$–$N_3$** | **UiO@$PO_3$–(PEG)$_{20}$–$N_3$** |
| 0 | 199 ± 6 | 218 ± 4 | 226 ± 5 |
| 1 | n.a. | 215 ± 6 | 225 ± 4 |
| 6 | n.a. | 210 ± 6 | 227 ± 4 |
| 24 | n.a. | 140 ± 12 | 230 ± 5 |
| 48 | n.a. | n.a. | 226 ± 5 |
| 96 | n.a. | n.a. | 224 ± 4 |
| 168 | n.a. | n.a. | 228 ± 6 |
| 336 | n.a. | n.a. | 223 ± 8 |

PXRD of the UiO@$PO_3$–(PEG)$_{20}$–$N_3$ particles after incubation in PBS for one week served also to confirm the stabilization of UiO nanoparticles after their functionalization with the long PEG ligand (Figure S9).

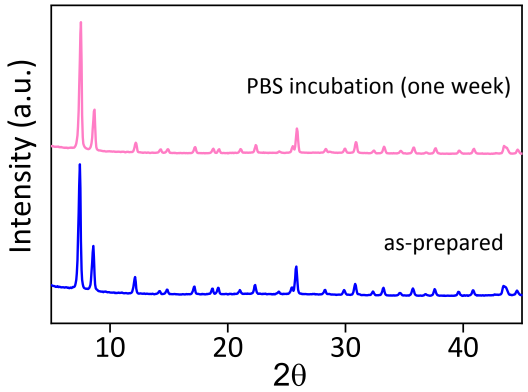


**Figure S9**. PXRD patterns of UiO@$PO_3$–$(PEG)_{20}$–$N_3$ particles as-prepared and after incubation in PBS (0.1 M, pH = 7.4) for 1 week.

As expected, the porosity of the UiO was affected by the PEG functionalization as shown in Figure 1H. The areas were calculated from the Barrett–Emmett–Teller ($S_{BET}$) method; Figure S10 show the linear region for the BET equation with P/Po between 0.05 and 0.2. Micropore volume ($V_{micro}$) was calculated by the t-plot method. Taking into account that the PEG part in the UiO@$PO_3$–$(PEG)_{20}$–$N_3$ particles accounts for about 42.6 wt % as determined by HPLC, their textural data were corrected considering exclusively the UiO-66 weight (Table S5).

**Table S5**. Textural properties of UiO and UiO@$PO_3$–$(PEG)_{20}$–$N_3$ particles.

| **Sample** | **$S_{BET}$ ($m^2 \cdot g^{-1}$)** | **$V_{micro}$ ($cm^3 \cdot g^{-1}$)** | **$S_{BET}$ ($m^2 \cdot g^{-1}$)*** | **$V_{micro}$ ($cm^3 \cdot g^{-1}$)*** |
|---|---|---|---|---|
| UiO | 1299.6 | 0.510 | – | – |
| UiO@$PO_3$–$(PEG)_{20}$–$N_3$ | 511.5 | 0.193 | 891.1 | 0.336 |

*corrected considering exclusively the UiO-66 weight as determined by HPLC.

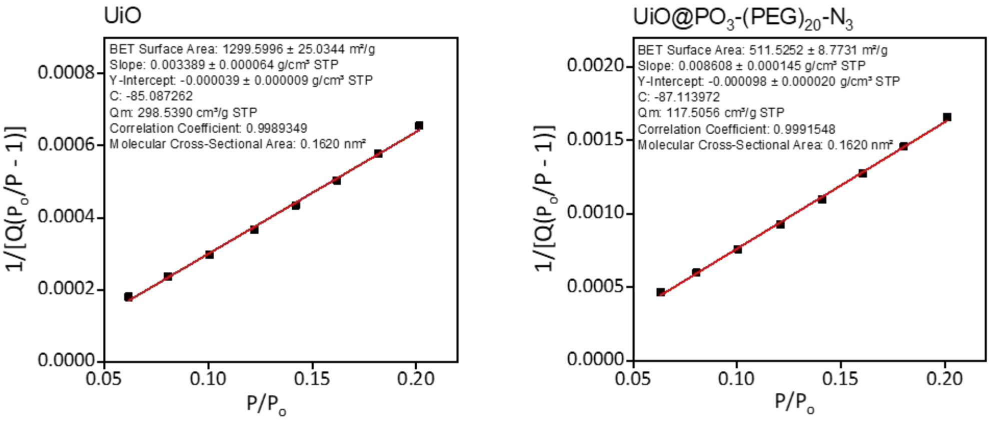


**Figure S10**. Plot of the linear region for the BET equation with P/Po between 0.05 and 0.2 of the experimental $N_2$ isotherms obtained with UiO and UiO@$PO_3$–$(PEG)_{20}$–$N_3$ particles.

The interaction between the $N_3$–PEG–$PO_3$ ligands and the UiO nanoparticles was investigated by comparison of $^1H$ and $^{31}P$ NMR spectra of the UiO@$PO_3$–$(PEG)_{20}$–$N_3$ particles, the resulting mixture after digestion of the particles with NaOH, and the free ligands. The results (Figure S11) showed that the terminal –$PO_3$ groups of the PEG molecules were coordinated with the Zr sites at the surface of the UiO particles.

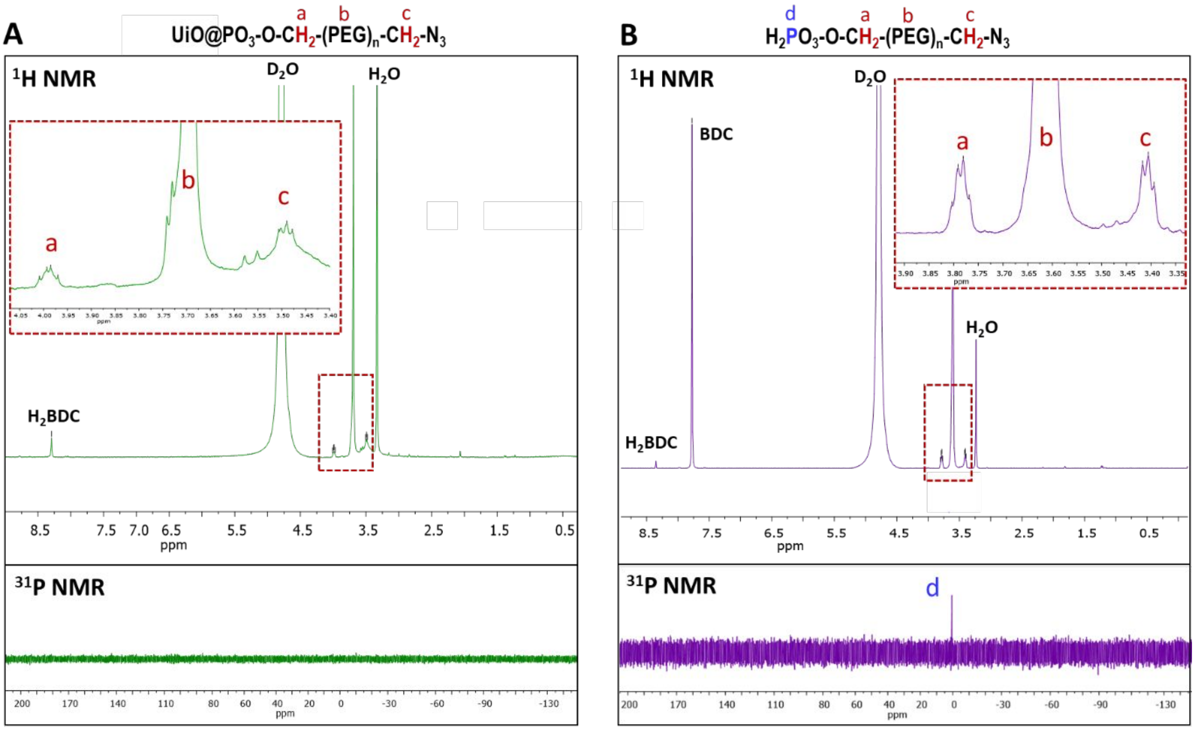


**Figure S11.** $^1H$ and $^{31}P$ NMR spectra of (A) UiO@$PO_3$–$(PEG)_{20}$–$N_3$ particles dispersed in $D_2O$, and (B) the resulting mixture solution after digestion of the particles.

## S5. Doxorubicin loading and characterization

DOX loading procedure: UiO nanoparticles were loaded with doxorubicin (DOX, selected as model of antitumoral drug and because it can be easily quantified by UV-Vis spectroscopy) just by mixing the UiO particles as dispersed in MeOH (3 mL, 20 mg/mL) with a solution of DOX in MeOH (3 mL, 10 mg/mL), having thus 0.5 g of DOX per mg of MOF during the incubation. The mixture was incubated overnight at RT to ensure that the maximum loading was reached. Next day, the DOX@UiO particles were collected by centrifugation, washed twice with MeOH in order to remove the DOX weakly adsorbed onto the UiO surface, and redisperse in MeOH for further use.

Quantification by UV-Vis: The amount of DOX loaded on the UiO particles was quantified indirectly, by measuring by UV-Vis the DOX remaining in the supernatant after centrifugation and washing steps of the DOX@UiO particles. The DOX concentration in the supernatants was determined by interpolation of the measured absorbance at the maximum absorption peak (480 nm) to a previously constructed analytical calibration curve (Figure S12). Additionally, we measured the amount of DOX released during the purification and washing steps in the following functionalization of the DOX@UiO with the PEG ligands, being such amount negligible (of only 0.12 wt%).

The loading efficiency (LE) was calculated using the following equation:

$$LE\ (\%) = \frac{mg\ DOX_{loaded}}{mg\ DOX_{added}} x\ 100 = \frac{mg\ DOX_{added} - mg\ DOX_{non-loaded}}{mg\ DOX_{added}} x\ 100$$

The loading capacity (LC) was calculated using the following equation:

$$LC\ (wt\%) = \frac{mg\ DOX_{loaded}}{mg\ MOF} x\ 100$$

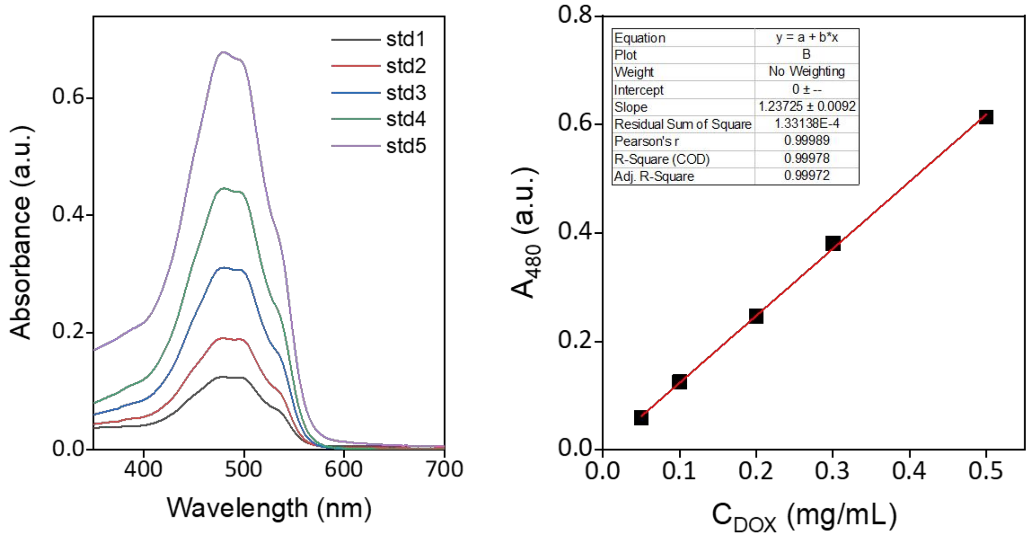


**Figure S12**. Calibration curve of DOX in MeOH, sowing the UV-Vis spectra of the different prepared DOX standards, and the plot of the absorbance at the maximum absorbance peak (480 nm) as a function of DOX concentration (in mg/mL). Calibration equation is obtained by fitting a linear regression line to the collected data.

Changes of the hydrodynamic size, surface charge of the UiO articles after the loading of the DOX molecules were measured, as well as after their PEG functionalization, see Table S6 and Figure S13. The textural properties of these DOX loaded particles after PEG functionalization were also determined, and taking into account that this is a gravimetric technique, values were corrected by subtracting the contribution from non-porous DOX mass (5.4 wt%) as shown in Table S7 particles after correction considering exclusively the UiO@$PO_3$–PEG–$N_3$ weight.

**Table S6**. Hydrodynamic size (mean value ± SD), polydispersity index (PDI), and ζ-potential (mean value ± SD) of DOX@UiO and DOX@UiO@$PO_3$–PEG–$N_3$ particles as dispersed in Milli-Q water. Values of UiO and UiO@$PO_3$–$(PEG)_{20}$–$N_3$ particles are also shown for comparison.

| Sample | Size (nm) | PDI | ζ-potential (mV) |
|---|---|---|---|
| UiO | 198 ± 4 | 0.024 | -14.8 ± 0.8 |
| DOX@UiO | 204 ± 3 | 0.042 | -12.9 ± 1.0 |
| UiO@$PO_3$–$(PEG)_{20}$–$N_3$ | 224 ± 5 | 0.083 | -22.0 ± 1.2 |
| DOX@UiO@$PO_3$–PEG–$N_3$ | 223 ± 7 | 0.090 | -20.8 ± 1.3 |

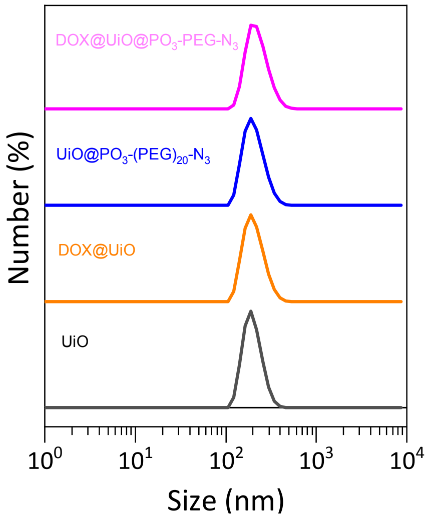


**Figure S13**. DLS size distributions by number of the different as-prepared MOF particles in Milli-Q water. Note that the dispersions of the particles in water is done just prior to the DLS measurements.

**Table S7**. Textural properties of DOX@UiO@$PO_3$–PEG–$N_3$ particles. Values corresponding to UiO@$PO_3$–$(PEG)_{20}$–$N_3$ are also shown for comparison

| Sample | $S_{BET}$ ($m^2 \cdot g^{-1}$) | $V_{micro}$ ($cm^3 \cdot g^{-1}$) | $S_{BET}$ ($m^2 \cdot g^{-1}$)* | $V_{micro}$ ($cm^3 \cdot g^{-1}$)* |
|---|---|---|---|---|
| UiO@$PO_3$–$(PEG)_{20}$–$N_3$ | 511.5 | 0.193 | – | – |
| DOX@UiO@$PO_3$–PEG–$N_3$ | 470.0 | 0.182 | 496.3 | 0.192 |

*corrected considering exclusively the UiO@$PO_3$–$(PEG)_{20}$–$N_3$ weight.

## S6. Enzyme-triggered release studies

*In vitro* release of DOX from MOF nanoparticles under optimized conditions: DOX@UiO and DOX@UiO@$PO_3$–PEG–$N_3$ particles were incubated with ALP to investigate the release rate of DOX in the presence of the enzyme in both cases. Each MOF solution (1 mL, 5 mg/mL, dispersed in HEPES buffer 0.01 M at pH = 7.2 and containing 2 mM of $Mg^{+2}$) was placed in an epperdorf vial and the ALP solution was added (50 µL, 20 U/mL), being thus the final concentration of ALP of *ca.* 1 U/mL. Controls were performed without adding ALP. The vials were incubated in a water bath at 37 °C for different times. After each time, vials were centrifugated to separate the MOF particles (pellets) and the supernatants (containing the DOX released and the PEG cleaved) were taken and measured by both HPLC-MS and UV-Vis spectroscopy. The amount of DOX released at each time was quantified by using previously obtained UV-Vis and HPLC calibration curves (using standards of known concentrations analysed by both techniques). The amount of PEG cleaved at each time was also quantified by HPLC. The total contents of DOX and PEG in the MOF particles was determined by analysing the resulting mixture after digestion of the particles with NaOH. Experiments at each incubation time were done in triplicate, and the percentages of DOX release and PEG release were calculated using the following equations:

$$DOX\ released\ (\%) = \frac{mg\ DOX_{released}}{mg\ DOX_{total}} x\ 100$$

$$PEG\ cleaved\ (\%) = \frac{mg\ PEG_{cleaved}}{mg\ PEG_{total}} x\ 100$$

The obtained release profiles are plotted in Figure 2D, showing significant differences for DOX@UiO and DOX@UiO@$PO_3$–PEG–$N_3$ particles that clearly indicated the protective effect of the PEG coating to avoid the premature drug release, as well as the role of ALP as a trigger to promote the PEG cleavage and subsequent drug release.

pH-dependant DOX release from MOF particles: The effect of the pH on the DOX release from DOX@UiO@$PO_3$–PEG–$N_3$ particles was studied in PBS at three different pH values (pH=5.5, 6.5 and 7.4). The DOX release over time at each pH was monitored by UV-Vis spectroscopy, using the same protocol as described above for the release study in the presence of the enzyme. Experiments were done in triplicate. The percentages of DOX released were calculated as indicated above, and results are presented in Figure S14. The DOX release profiles in PBS at pH=7.4 and HEPES at pH=7.2 were very similar, revealing that the composition of the buffer solution did not affect to the integrity of the PEGylated particles in the long term (i.e., after 72 h of incubation), and thus confirming again that the PEG coating was able to stabilize the DOX@UiO particles in PBS, that is, avoiding the attack of phosphate ions to the Zr sites on the UiO surface.

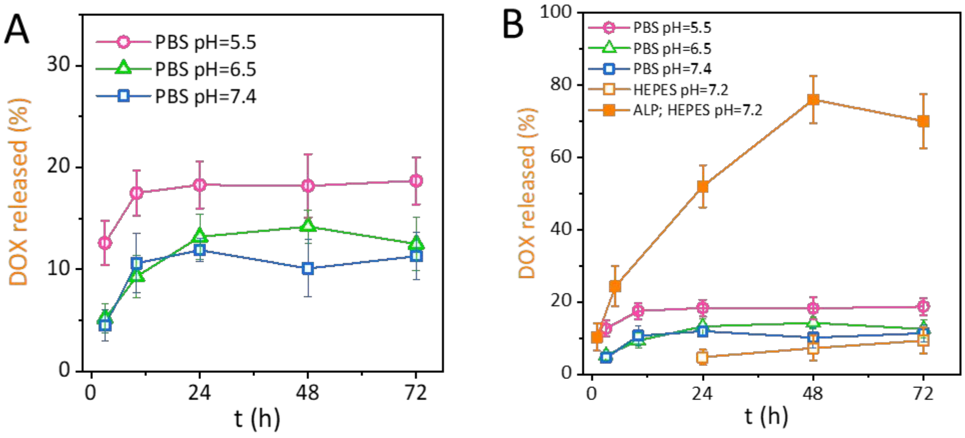


**Figure S14.** (A) Kinetic profiles of DOX released from the MOF particles (5 mg/mL) incubated in PBS buffer solutions at different pH values (pH=5.5, 6.5 and 7.4). (B) Comparison with the release profile of DOX when the DOX@UiO@$PO_3$–PEG–$N_3$ particles were incubated in HEPES buffer at pH = 7.2 in the absence and presence of the ALP enzyme (1 U/mL).

Investigation of the cleavage site of the PEG by the enzyme via HLPC-MS analysis

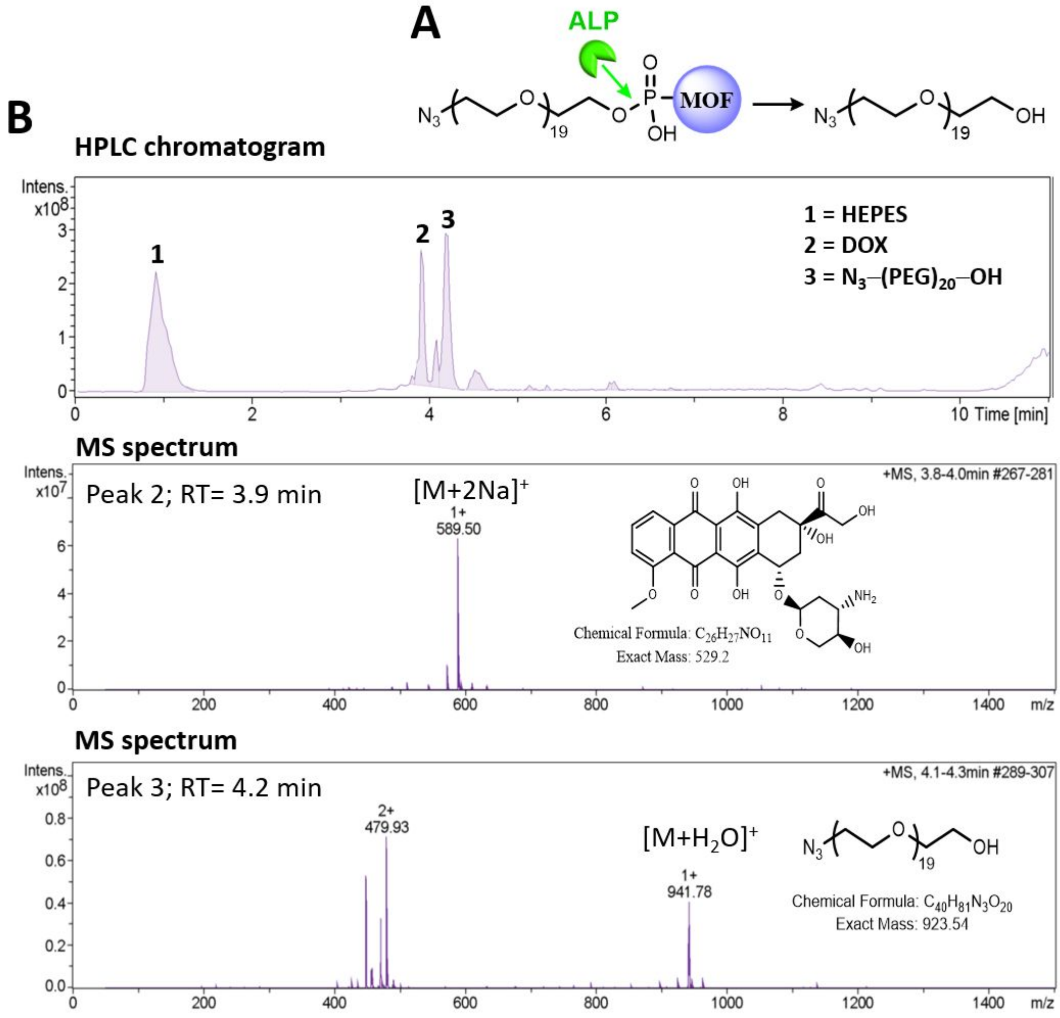


**Figure S15**. (A) Cleavage site of the DOX@UiO@$PO_3$–PEG–$N_3$ particles by ALP enzymolysis, on the basis of the MS data. (B) HPLC chromatogram and MS spectra (in positive ion mode) of the corresponding peaks obtained after 5 h of incubation of MOF particles (5 mg/mL) with ALP (1 U/mL) in HEPES buffer (0.01 M, 2 mM $Mg^{+2}$, pH = 7.2) at 37 ºC.

## S7. Click chemistry on MOF nanoparticles

Click-chemistry procedure: DOX@UiO@$PO_3$–PEG–$N_3$ particles were labelled with a fluorescent probe, specifically sulfo-cyanine3-azodibenzocyclooctyne (referred to as Cy3-DBCO), by using a microwave-assisted mehod. Briefly, the DOX@UiO@$PO_3$–PEG–$N_3$ particles dispersed in water (1.5 mL, 20 mg/mL) were mixed with an aqueous solution of the dye (1.5 mL, 10 µM, i.e., 9.55 µg/mL) in a 5-mL microwave vial. The reaction was performed at 80 °C for 30 min (14 W), in an Initiator Classic microwave reactor from Biotage. Next, the DOX@UiO@$PO_3$–PEG–Cy3 particles were purified by centrifugation, washed twice with water to remove the dye weakly adsorbed on the MOF surface, and finally redisperse in water.

Quantification by UV-Vis: The amount of Cy3 attached to the MOF particles was quantified indirectly, by measuring by UV-Vis the Cy3-DBCO remaining in the supernatant after centrifugation and washing steps of the particles. The Cy3-DBCO concentration in the supernatants was determined by interpolation of the measured absorbance at the maximum absorption peak (550 nm) to a previously constructed analytical calibration curve (Figure S16). The reaction efficiency was very high (98 %), whereas the amount of Cy3 attached as determined by UV-Vis was found to be 3.5 wt%.

The "click" reaction efficiency (CE) was calculated using the following equation:

$$CE\ (\%) = \frac{mg\ Cy3_{attached}}{mg\ Cy3_{added}} x\ 100 = \frac{mg\ Cy3_{added} - mg\ Cy3_{non-attached}}{mg\ Cy3_{added}} x\ 100$$

The Cy3 content in the MOF particles was calculated using the following equation:

$$Cy3\ (wt\%) = \frac{mg\ Cy3_{attached}}{mg\ MOF} x\ 100$$

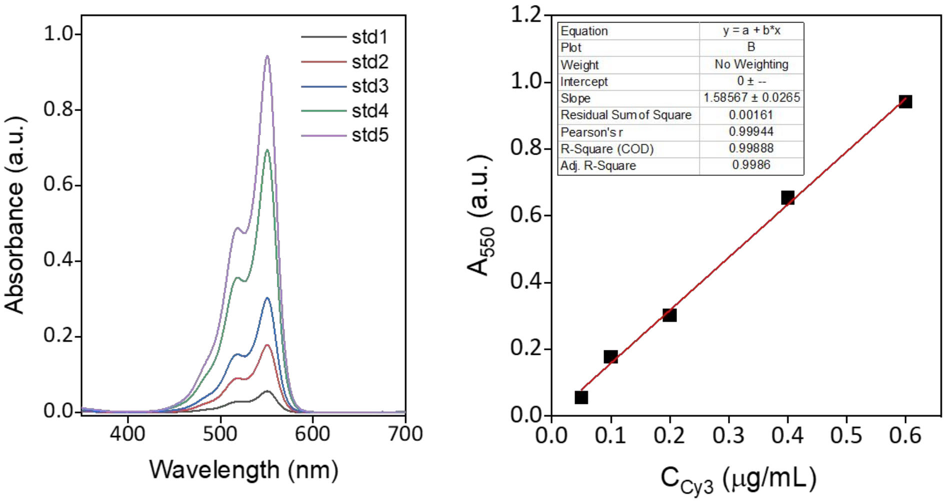


**Figure S16**. Calibration curve of Cy3-DBCO in water, showing the UV-Vis spectra of the different prepared Cy3-DBCO standards, and the plot of the absorbance at the maximum absorbance peak (550 nm) as a function of Cy3-DBCO concentration (in µg/mL). Calibration equation is obtained by fitting a linear regression line to the collected data.

The as-prepared DOX@UiO@$PO_3$–PEG–Cy3 particles were characterized by DLS and LDA (Table S8), observing just a quite small increase in the hydrodynamic size (about 4 nm) and in the negative surface charge after the dye binding; it must be noted that such increments are too small to be significant taking into account the broad DLS profile of the particles (shown in Figure S17).

**Table S8**. Hydrodynamic size (mean value ± SD), polydispersity index (PDI), and ζ-potential (mean value ± SD) of DOX@UiO@$PO_3$–PEG–Cy3 particles as dispersed in Milli-Q water. Values corresponding to DOX@UiO@$PO_3$–PEG–$N_3$ particles are also shown for comparison.

| Sample | Size (nm) | PDI | ζ-potential (mV) |
|---|---|---|---|
| DOX@UiO@$PO_3$–PEG–$N_3$ | 223 ± 7 | 0.090 | -20.8 ± 1.3 |
| DOX@UiO@$PO_3$–PEG–Cy3 | 227 ± 6 | 0.087 | -25.2 ± 1.0 |

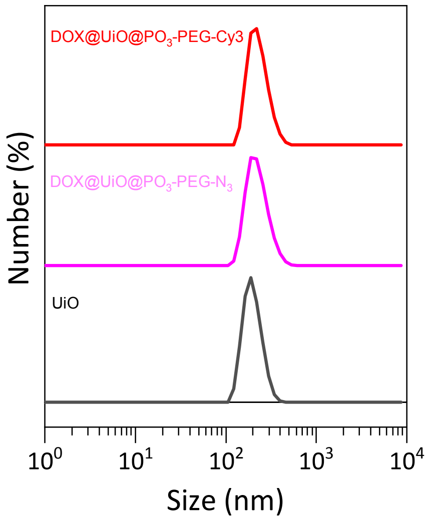


**Figure S17**. DLS size distributions by number of the as-prepared DOX-loaded MOF particles before and after the attachment of the fluorescent Cy3 probe. The pristine UiO particles are also shown for comparison.

Quantification by TGA: To avoid the contribution of the DOX content in the TGA analysis, the quantification of the Cy3 content attached to the UiO@$PO_3$–PEG–$N_3$ particles was carried out using MOF particles without DOX loaded. The Cy3-modified MOF particles presented a very similar thermal stability as the non-modified ones as determined by TGA (Figure S18), confirming that the MOF structure was not compromised during the SPAAC reaction by MW. The 100% mass of each material was normalised to 150°C to avoid taking solvent evaporation into account of organic mass loss. A small organic mass addition (quantified as 3.2 wt%) was obtained, indicating the successful incorporation of Cy3 on the particles.

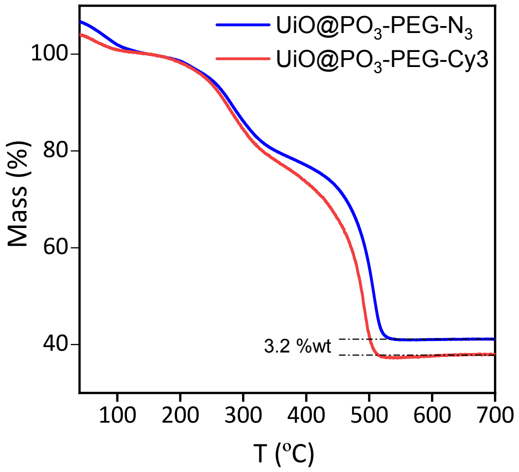


**Figure S18**. TGA curves of the UiO@$PO_3$–PEG–Cy3 particles and the corresponding non-modified counterparts, UiO@$PO_3$–PEG–$N_3$ particles.

<u>Stability of Cy3-labelled MOF particles in cell culture media</u>: UiO@$PO_3$–PEG–Cy3 particles were incubated at 37 ºC with complete cell medium (i.e., DMEM without phenol red supplemented with 10% v/v FBS, 100 units/mL penicillin, and 100 µg/mL streptomycin) for different times. After specific times (3h, 10h, 24h and 48 h), the MOF particles were separated by centrifugation and the supernatants (containing the Cy3 released) were taken and measured by UV-Vis spectroscopy. The amount of Cy3 released over time was quantified by using a previously obtained UV-Vis calibration curve (using standards of known concentrations prepared in complete cell media, Figure S19). The total content of Cy3 in the MOF particles was determined by analysing the resulting mixture after digestion of the particles with NaOH. Experiments at each incubation time were done in triplicate and the percentage of Cy3 released were calculated.

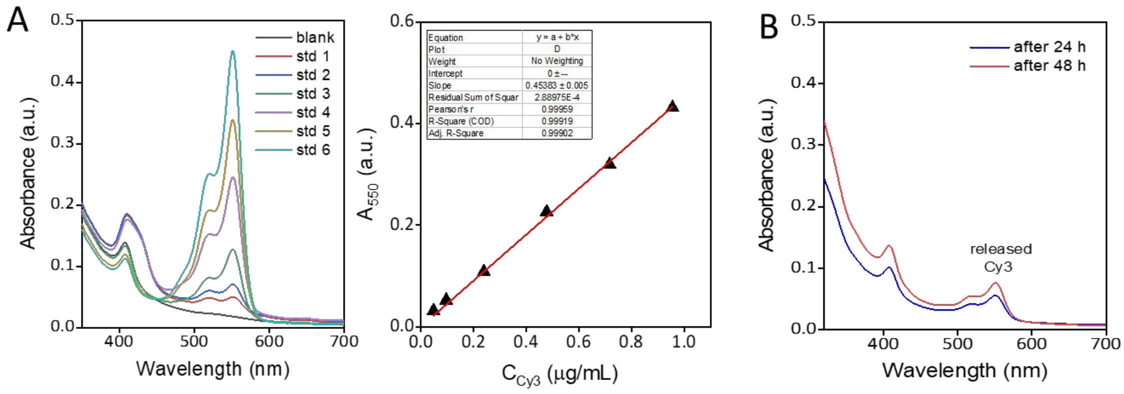


**Figure S19**. (A) Calibration curve of Cy3 in complete cell medium, showing the UV-Vis spectra of the different prepared standards with the maximum absorbance peak at 550 nm. (B) UV-Vis spectra of the Cy3 released from the UiO@$PO_3$–PEG–Cy3 particles after being incubated in cell media at 37 ºC for 24 h and 48 h.

## S8. Cell studies

Doubly fluorescent-labelled MOF, having fluorescein (F) loaded on the UiO and Cy3 attached to the PEG ligands on the particle surface, were prepared to easily monitor the particles by confocal microscopy and flow cytometry. Fluorescent F@UiO particles were used as control particles to study the effect of the PEG coating on the cargo release kinetics. The amount of fluorescein loaded on the UiO particles and after PEGylation of the particles was quantified by UV-Vis, in the same way as described above for quantifying the DOX amount. The content of F in the F@UiO and F@UiO@PEG was found to be 7.7 wt% and 7.4 wt%, respectively. The amount of PEG-Cy3 in the F@UiO@PEG-Cy3 was 37.9 wt% as measured by ICP-OES.

The cytotoxicity of the different prepared MOF nanoparticles was first evaluated in order to select the appropriate MOF concentrations for the further cell uptake studies. Note that the MOF particles concentration $C_{MOF}$ refers to the concentration of UiO in mg/mL (considering exclusively the UiO weight). As shown in Figure S18, all MOF particles showed the typical dose-dependent response. The half-maximal effective concentrations ($EC_{50}$) were determined from these viability curves and are shown in Figure S20A. At a selected concentration of 0.2 mg/mL, we also investigated the toxicity in normal cells, specifically in human mesenchymal stem cells (MSC), and the results are shown in Figure S20B.

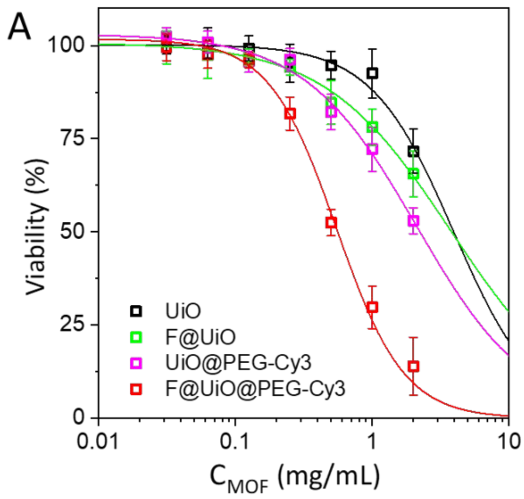


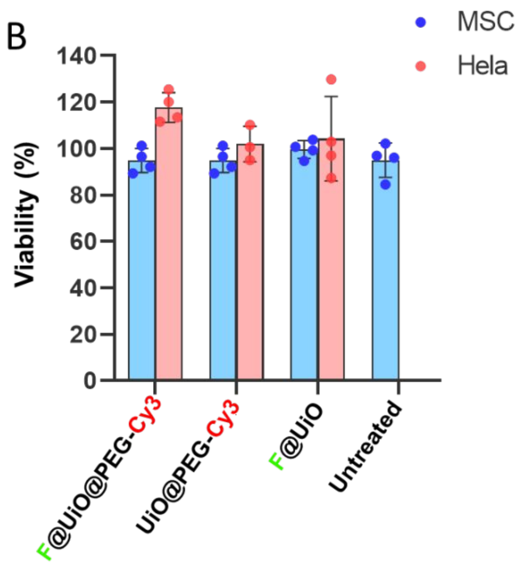


| Sample | $EC_{50}$ (mg/mL) |
|---|---|
| UiO | 3.93 ± 0.09 |
| F@UiO | 3.74 ± 0.08 |
| UiO@PEG-Cy3 | 2.11 ± 0.06 |
| F@UiO@PEG-Cy3 | 0.54 ± 0.02 |

**Figure S20**. (A) Cell viability using CellTiter-Glo assay (based on ATP quantification) of HeLa cells under 24 h exposure to increasing concentrations of the different prepared MOF particles Half-maximal responses ($EC_{50}$ values) were calculated by fitting (logistic function in OriginLab, fixing minimum viability to 0 %). (B) Cell viability using CellTiter-Glo assay of HeLa and MSC cells under 24 h exposure to the different prepared MOF particles at a concentration of $C_{MOF}$ = 0.2 mg/mL. $C_{MOF}$ corresponds to the mg/mL of UiO, that is considering exclusively the UiO weight.

Confocal microscopy images of HeLa cells incubated with the MOF particles at a concentration $C_{MOF}$ of 0.2 mg/mL were taken at different incubation times (3 h, 10 h and 24 h) as shown in Figure 3. Control experiments with equivalent amounts of free fluorescein and free PEG-Cy3 as present in the F@UiO@PEG-Cy3 particles were also performed to investigate the cellular uptake of such free fluorescent components (Figure S21).

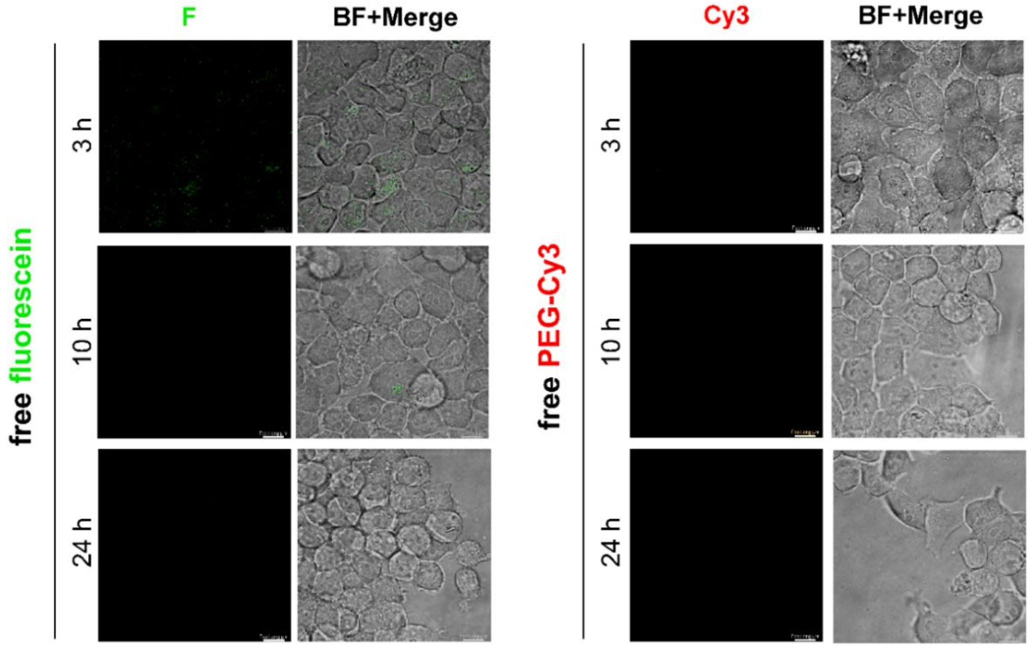


**Figure S21.** Confocal microscopy images of HeLa cells incubated with free fluorescein (F) and free PEG-Cy3 at concentrations equivalent as those present in the functionalized F@UiO@PEG-Cy3 particles (0.2 mg/mL) for 3 h, 10 h or 24 h. Green (F) and red (Cy3) fluorescence channels, merge and bright field (BF) are shown.

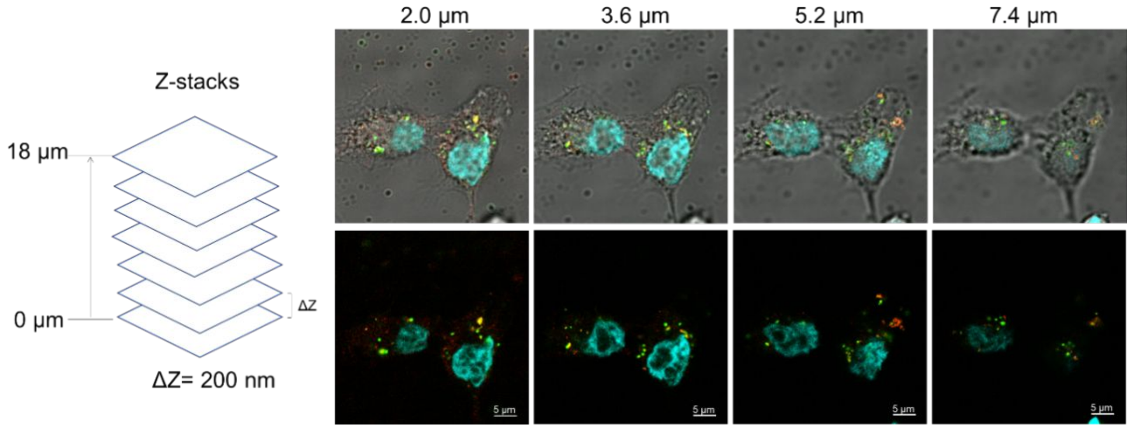


**Figure S22**. Confocal z-stack slices of HeLa cells incubated with F@UiO@PEG-Cy3 particles at 0.2 mg/mL for 3 h. Scheme with distance of the different Z stacks from the coverslip in micrometer (left). Green (F) and red (Cy3) fluorescence channels are merged; yellow marks indicate the colocalization between green and red channels. Bright field images (up) show the limitation of the cytoplasmic membrane. Nucleus is stained with DAPI (marked in blue).

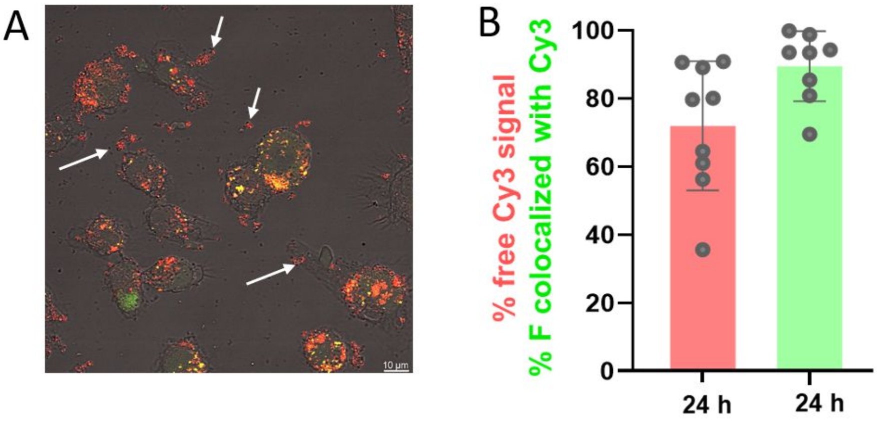


**Figure S23**. (A) Representative confocal image of HeLa cells incubated with F@UiO@PEG-Cy3 particles at 0.2 mg/mL for 24 h; white arrows indicate some PEG-Cy3 apparently detached. (B) Quantitative data of colocalization of Cy3 and fluorescein after 24 h.

The cellular uptake of the MOF particles was also investigated by flow cytometry (Figures S22-24), using the gating strategy shown in Figure S22. It is worth noting that flow cytometry histogram profiles obtained for F@UiO@PEG-Cy3 particles in HeLa cells were broader than those for naked F@UiO particles, which may represent the heterogeneity of ALP activity in the degradation of functionalized MOF particles at the single cell level.

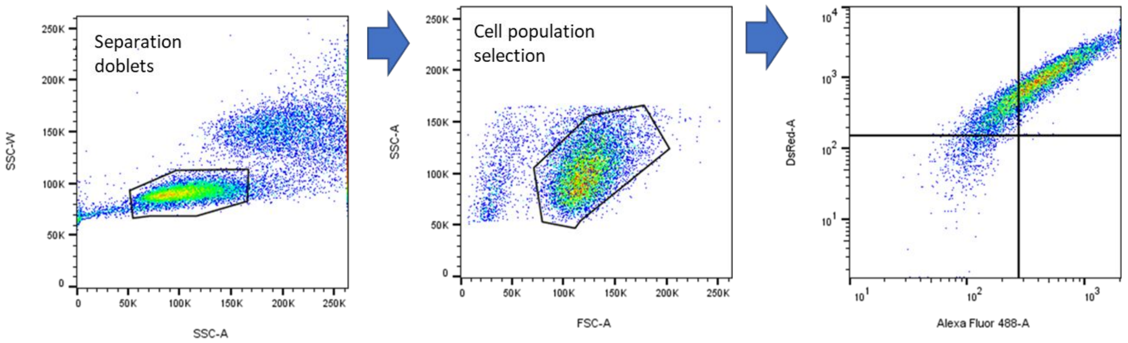


**Figure S24.** Gating strategy using as a representative example the sample of HeLa cells incubated with the F@UiO@PEG-Cy3 particles for 24 h. Two fluorescence channels were recorded, the green channel (named as Alexa Fluor 488 by the instrument) to the red channel (named as DsRED by the instrument) for detecting the Fluorescein and Cy3, respectively. >10000 events for each condition were collected.

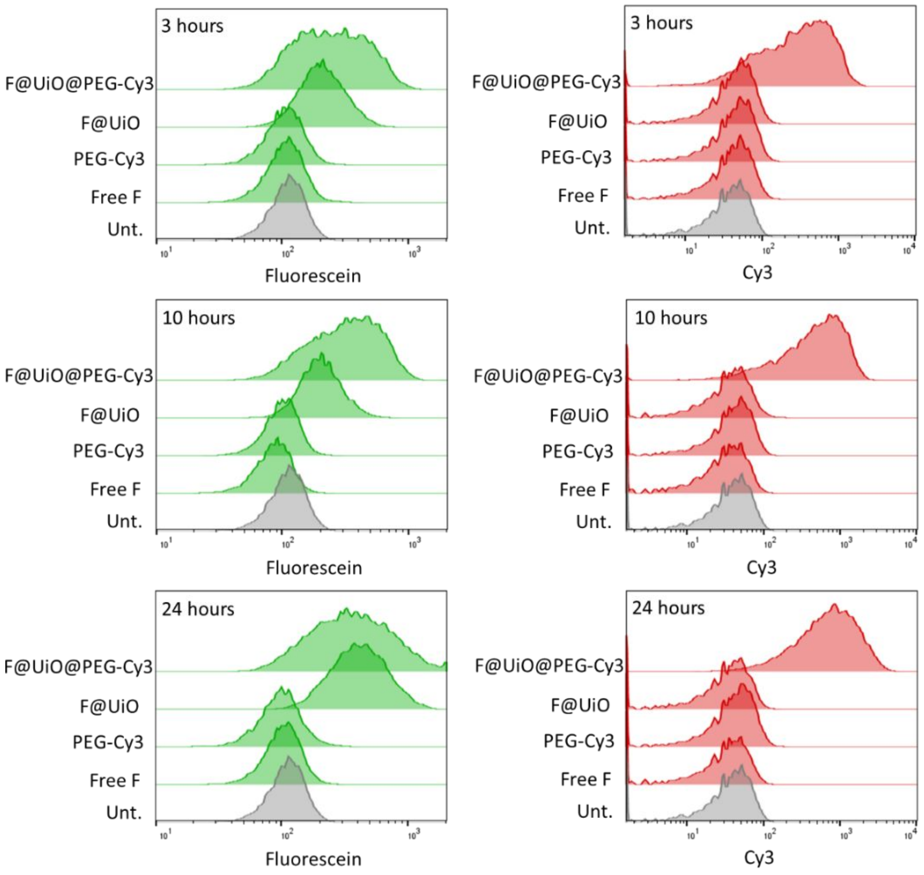


**Figure S25.** Cellular uptake studies by flow cytometry. HeLa cells were incubated with bare F@UiO and functionalized F@UiO@PEG-Cy3 particles at equivalent MOF concentrations. Control samples of free fluorescein and free PEG-Cy3 (at equivalent amounts as present in MOF particles), and untreated cells were also analysed. Histograms showing the intracellular levels of Fluorescein (green) and Cy3 (red channel). >10000 events for each condition were collected.

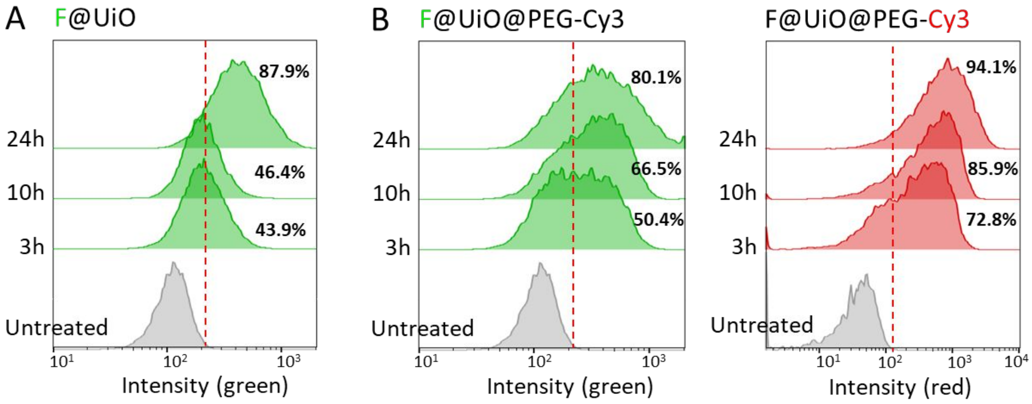


**Figure S26**. Flow cytometry fluorescence distribution histograms of HeLa cells treated with (A) bare F@UiO or (B) functionalized F@UiO@PEG-Cy3 particles (at 0.2 mg/mL; concentration considering exclusively the UiO weight) for different incubation times, 3 h, 10 h and 24 h. Green (Fluorescein) and red (Cy3) fluorescence channels are shown. The right-area from the red dotted line in each graph represents the sorting gate defined as positive event. The percentage of cells that fell into that window and were counted as positive events is provided.

## S9. References

(1) Gauthier, B. R.; Rubio-Contreras, D.; Gómez-Rosado. J. C.; Capitán-Morales, L. C.; Hmadcha, A.; Soria, B.; Lachaud, C. C. Human Omental Mesothelial Cells Impart an Immunomodulatory Landscape Impeding B- and T-Cell Activation. *Int. J. Mol. Sci.* **2022**, *23*, 5924.

(2) Markopoulou, P.; Panagiotou, N.; Li, A.; Bueno-Perez, R.; Madden, D., Buchanan, S.; Fairen-Jimenez, D.; Shiels, P. G.; Forgan, R. S. Identifying differing intracellular cargo release mechanisms by monitoring in vitro drug delivery from MOFs in real time. *Cell Rep. Phys. Sci.*, **2020**, *1*, 100254.
